# Recent progress in metal oxide-based electrode materials for safe and sustainable variants of supercapacitors

**DOI:** 10.3389/fchem.2024.1402563

**Published:** 2024-05-20

**Authors:** Ali Asghar, Karim Khan, Othman Hakami, Waleed M. Alamier, Syed Kashif Ali, Taharh Zelai, Muhammad Shahid Rashid, Ayesha Khan Tareen, Enaam A. Al-Harthi

**Affiliations:** ^1^ Additive Manufacturing Institute, Shenzhen University, Shenzhen, China; ^2^ Department of Physical Sciences, Chemistry Division, College of Science, Jazan University, Jazan, Saudi Arabia; ^3^ Department of Physical Sciences, Physics Division, College of Science, Jazan University, Jazan, Saudi Arabia; ^4^ School of Mechanical Engineering, Dongguan University of Technology, Dongguan, China; ^5^ College of Science, Department of Chemistry, University of Jeddah, Jeddah, Saudi Arabia

**Keywords:** electrochemical energy storage devices, selection of electrode material (metal oxide), synthesis methods, and transportation system, supercapacitor

## Abstract

A significant amount of energy can be produced using renewable energy sources; however, storing massive amounts of energy poses a substantial obstacle to energy production. Economic crisis has led to rapid developments in electrochemical (EC) energy storage devices (EESDs), especially rechargeable batteries, fuel cells, and supercapacitors (SCs), which are effective for energy storage systems. Researchers have lately suggested that among the various EESDs, the SC is an effective alternate for energy storage due to the presence of the following characteristics: SCs offer high-power density (PD), improvable energy density (ED), fast charging/discharging, and good cyclic stability. This review highlighted and analyzed the concepts of supercapacitors and types of supercapacitors on the basis of electrode materials, highlighted the several feasible synthesis processes for preparation of metal oxide (MO) nanoparticles, and discussed the morphological effects of MOs on the electrochemical performance of the devices. In this review, we primarily focus on pseudo-capacitors for SCs, which mainly contain MOs and their composite materials, and also highlight their future possibilities as a useful application of MO-based materials in supercapacitors. The novelty of MO’s electrode materials is primarily due to the presence of synergistic effects in the hybrid materials, rich redox activity, excellent conductivity, and chemical stability, making them excellent for SC applications.

## 1 Introduction

In today’s world, first, the demand for energy is higher than ever, and the need to reduce reliance on fossil fuels is becoming more urgent and critical (Khan et al., 2019a; Khan et al., 2020a; Khan et al., 2020b). Second, the challenge lies in storing renewable energy and ensuring its availability on demand (Smith et al., 2015; Khan et al., 2021a). Therefore, energy storage devices (ESDs) like batteries and supercapacitors are attractive alternatives to fossil fuels because they are much cleaner, eco-friendly, and more efficient energy storage systems (Velez-Fort et al., 2012; Smith et al., 2015; Khan et al., 2018a; Khan et al., 2018b; Dai et al., 2018; Khan et al., 2019a; Khan et al., 2019b; Khan et al., 2019c; Khan et al., 2019d; Khan et al., 2019e; Khan et al., 2019f; Tareen et al., 2019; Zhang et al., 2019; Chen et al., 2020a; Khan et al., 2020a; Shi Z. et al., 2020; Khan et al., 2020b; Khan et al., 2020c; Hu et al., 2020; Ahmad et al., 2021; Khan et al., 2021a; Wang L. et al., 2021; Khan et al., 2021b; Khan et al., 2021c; Khan et al., 2021d; Tareen et al., 2021; Wu et al., 2021; Cao et al., 2022; Tareen et al., 2022; Khan et al., 2023). These ESDs can store renewable energy for a long time and can provide steady, reliable energy at any moment (Zhang et al., 2019; Cao et al., 2022). These renewable energy sources are ideal for transportation, electric vehicles, and other energy storage applications (Şahin et al., 2022). Traditional batteries are the leading energy storage option but have some limitations, such as low power presentation, low cyclic life stability, and fear of explosion at high temperatures due to liquid electrolytes (Nan et al., 2019). Due to some restrictions on some types of batteries, the researcher community is finding better alternatives for energy storage that can be fulfilled by using a supercapacitor (SC). SCs, also known as ultracapacitors or electrochemical (EC) capacitors, are ESDs that bridge the gap between batteries and capacitors. They can store and supply energy much more rapidly than batteries and have a higher power density (PD). There are several forms of SCs based on their construction and usage of electrode materials (Nan et al., 2019; Şahin et al., 2022). The common types of SCs include electric double-layer capacitors (EDLCs), pseudo-capacitors (PCs), symmetric supercapacitors (SSCs), asymmetric supercapacitors (ASSCs), hybrid supercapacitors (HSCs), and nanostructured supercapacitors (NSSCs) (Chen et al., 2011; Lu et al., 2014; Ibrahim et al., 2016; Shao et al., 2018; Goswami et al., 2023). Different forms of electrode materials are employed in SCs. The most common electrode materials are perovskite materials (Nan et al., 2019), metal oxides (Liu et al., 2020; Liang et al., 2021; Khawar et al., 2022), metal phosphate (Asghar et al., 2023a), metal sulfides (Pothu et al., 2021; Asghar et al., 2023b), metal organic frameworks (MOFs) (Hamza et al., 2023a; Hamza et al., 2023b; Asghar et al., 2024), and conducting polymers (CPs) that have gained attention due to the incomparable properties of electrode materials, such as conductivity, precise capacity, small electronegativity, remarkable shape of the crystal, and redox activity. These properties make them ideal for many applications because SCs deliver energy quickly and efficiently. Their low cost and easy fabrication make them viable for large-scale production and application (Theerthagiri et al., 2020; Pothu et al., 2021; Asghar et al., 2022a; Khawar et al., 2022; Asghar et al., 2023a). In the past few years, researchers have been investigating electrode materials as well as improving the preparation methods to enhance the EC presentation of the electrode for more effectively storing larger quantities of energy.

On the other hand, renewable energy sources produce a large amount of energy, so these sources require efficient and reliable ESDs. ESDs are more attractive devices for energy storage due to their benefits such as their low cost, flexible capacity, and high efficiency. For this purpose, SCs, recognized as EESDs, store and release energy electrostatically, permitting them to supply high PD and good cycle life stability. A supercapacitor contains two electrodes, generally made of carbon-based materials, with a small separation. The basic electrostatic charge storage mechanism is that when voltage is applied to the electrode of an SC, the electrolytic ions in the solution are transferred to the opposite electrodes and create a dual layer of charge on the surface of both electrodes (Najib and Erdem, 2019; Dhapola et al., 2022). The key aspects that contribute to the high energy storage capacity of SCs are the large surface area (which can be gained by using porous materials) and low electrolyte separation (which causes easy movement of ions). In a simple capacitor, the capacitance (C = ε_o_ε_r_d/A) of the capacitor can be varied by changing the area of the plate (A), the distance between the plates, or the dielectric medium between the plates (ε_r_). Furthermore, the SC followed the same concept as conventional capacitors but employed an electrolyte material instead of a dielectric material (ε_r_) (Wasterlain et al., 2006). SCs have some benefits when compared to other ESDs, such as good PD, a rapid charging and discharging process, a wide range of temperatures, an energy flow cutoff after maximum charging, small equivalent series resistance in devices, an extendable lifetime, and being environmentally friendly (Glavin and Hurley, 2012; Jing et al., 2017; Ahmed et al., 2018).

**FIGURE 1:**
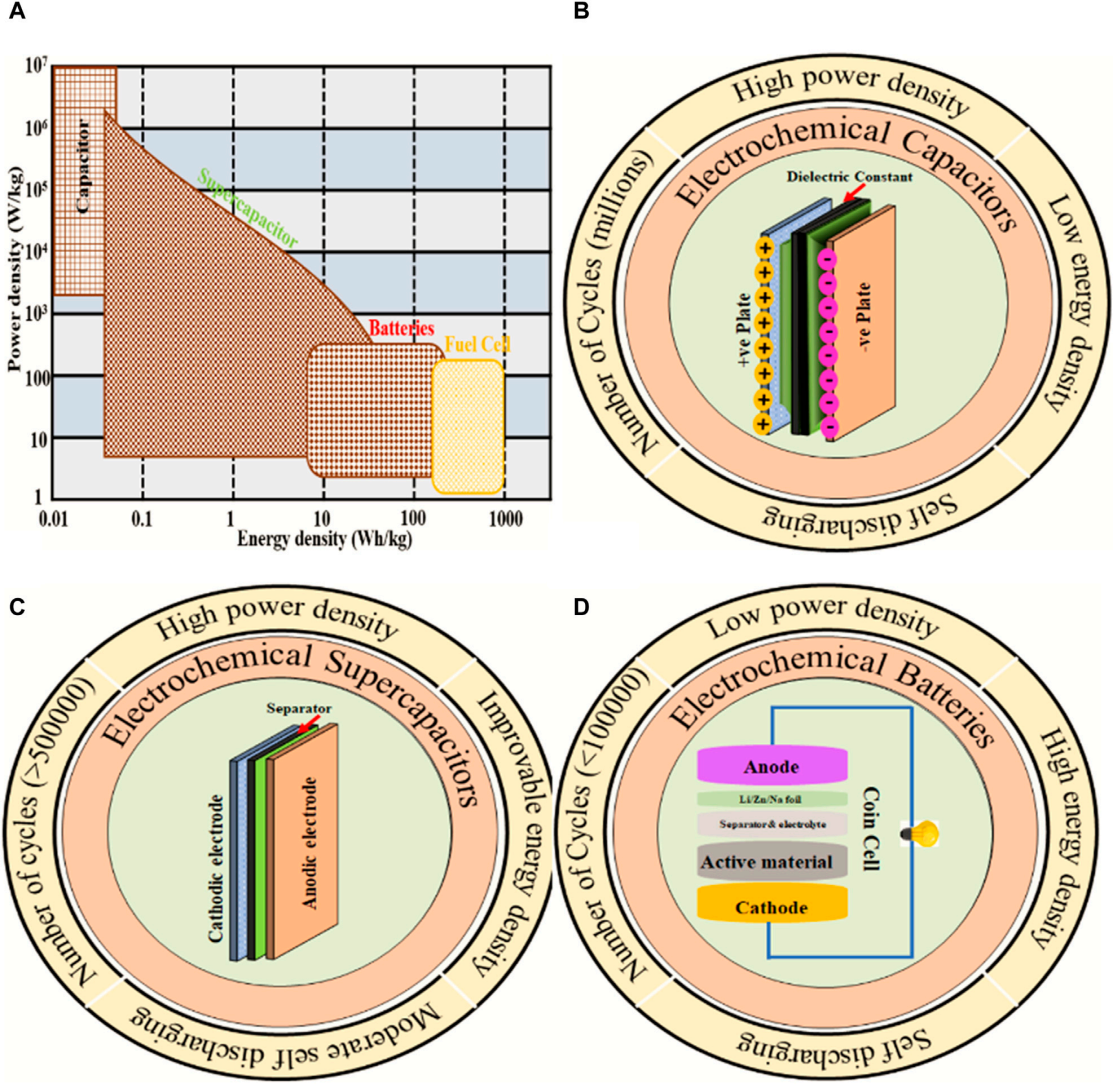
**(A)** Ragone plot of EESDs and electrochemical properties of **(B)** capacitors, **(C)** supercapacitors, and **(D)** batteries.

SCs have some limitations as compared to other ESDs, such as low operating efficiency, low energy density (ED), the need for a balancing circuit in series connections, challenges in market delivery and pricing, and high dielectric absorption (Glavin and Hurley, 2012; Şahin and Blaabjerg, 2020; Şahin et al., 2022). Figure 1A represents the Ragone plot that shows the classification of electrochemical devices (capacitors, batteries, SCs, and fuel cells). Batteries are another class of ESDs that provide high ED, low power transport, good cycle life stability, high heat generation, increased time constant (in hours), and low self-discharging. On the other hand, SCs fill the space between the batteries (high-energy) and capacitors (high-power), which have high PD, good stability and rate competence, and consistency and are safe to operate, as shown in Figures 1B–D (Kumar A. et al., 2022).

**FIGURE 2:**
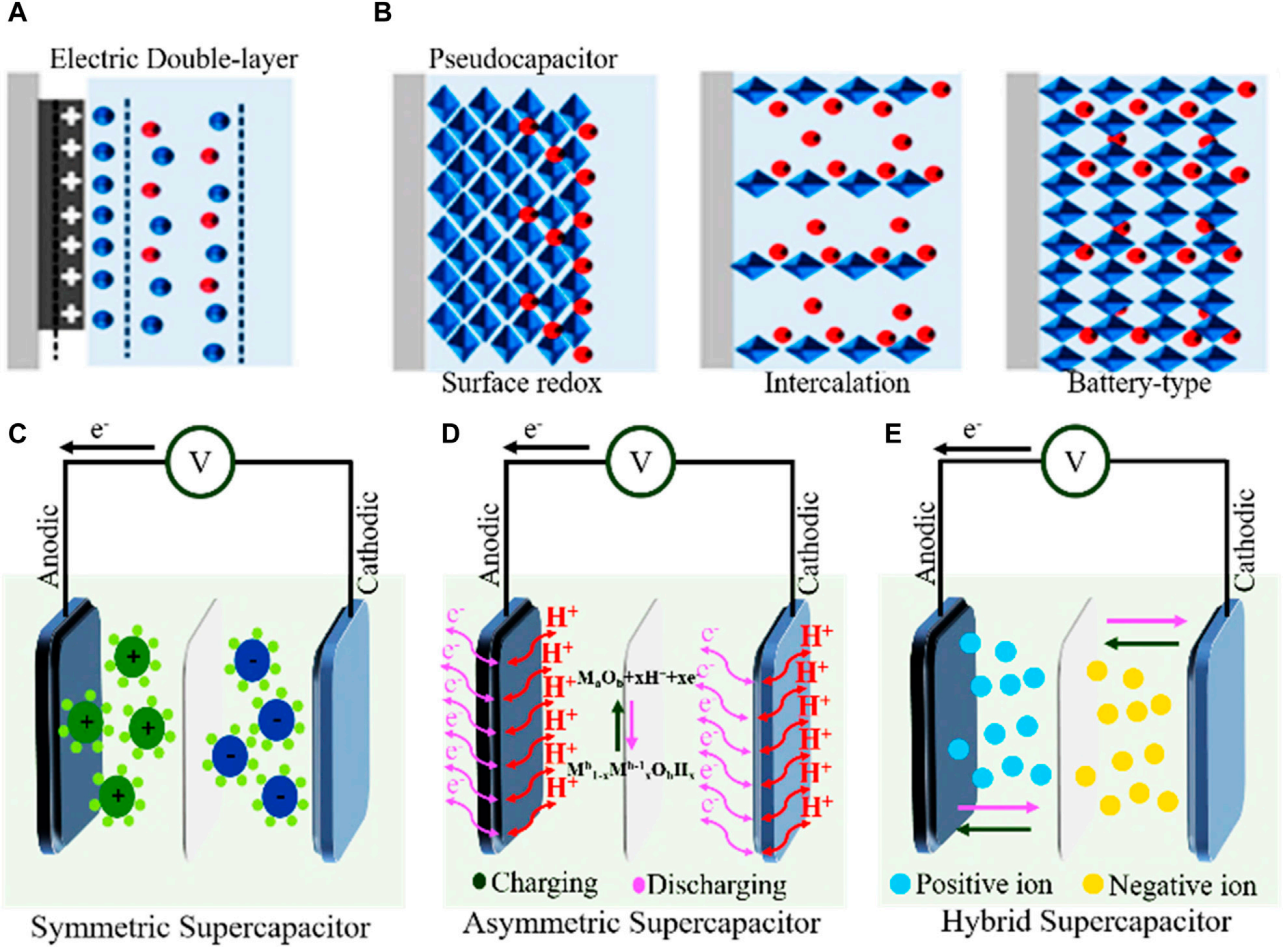
Types of supercapacitors: **(A)** EDLCs, **(B)** energy storage mechanisms of the pseudo-capacitor, **(C)** SSC, **(D)** ASSC, and **(E)** HSC (negative electrode battery-like and positive electrode an EDLC-like). Reproduced with permission from Zhang et al. (2022).

On the basis of charge storage mechanisms, SCs are classified into PCs, EDLCs, and HSCs (Zhang et al., 2022). The charge storage process in the EDLCs is accomplished by separating charges within the electrode material and electrolyte boundary, which causes physical adsorption where no other chemical reaction takes places on the surface of the electrode, as exhibited in Figure 2A. A pseudo-capacitor is a high-speed, superficial, and non-diffusion capacitor that stores charges by limiting the redox reaction to allow charges to stay stored and hence is also known as a redox capacitor. As shown in Figure 2B, PCs are classified into three types based on their mechanisms: surface redox PC, intercalation PC, and battery type PC. On the other hand, based on the composition of electrodes, SCs can be also classified into SSCs, ASSCs, and HSCs (Kumar S. et al., 2021).

SSCs are constructed by combining electrode materials of the same capacitance on the anode and cathode (the electrode is either based on an EDLC-based working mechanism or a PC-based working mechanism), as depicted in Figure 2C. Normally, SSCs consist of two similar electrode materials: carbon materials and pseudo-capacitance materials. However, ASSCs are constructed by combining two different electrode materials that possess an excellent potential window, as shown in Figure 2D. Generally, ASSCs consist of two dissimilar electrode materials: EDLC materials and PC materials. When a charge and discharge process occurs, the asymmetric SC is fully capable of utilizing the changed potential windows (PWs) within the electrodes for maximizing the working voltage of the SCs within a short period of time (Shao et al., 2018). Moreover, HSCs contain both battery-like negative electrodes that show EDLC insertion or conversion and capacitor-like positive electrodes that show physical adsorption, as shown in Figure 2E, and HSCs also have numerous advantages over other capacitors (Zhang et al., 2021).

**FIGURE 3:**
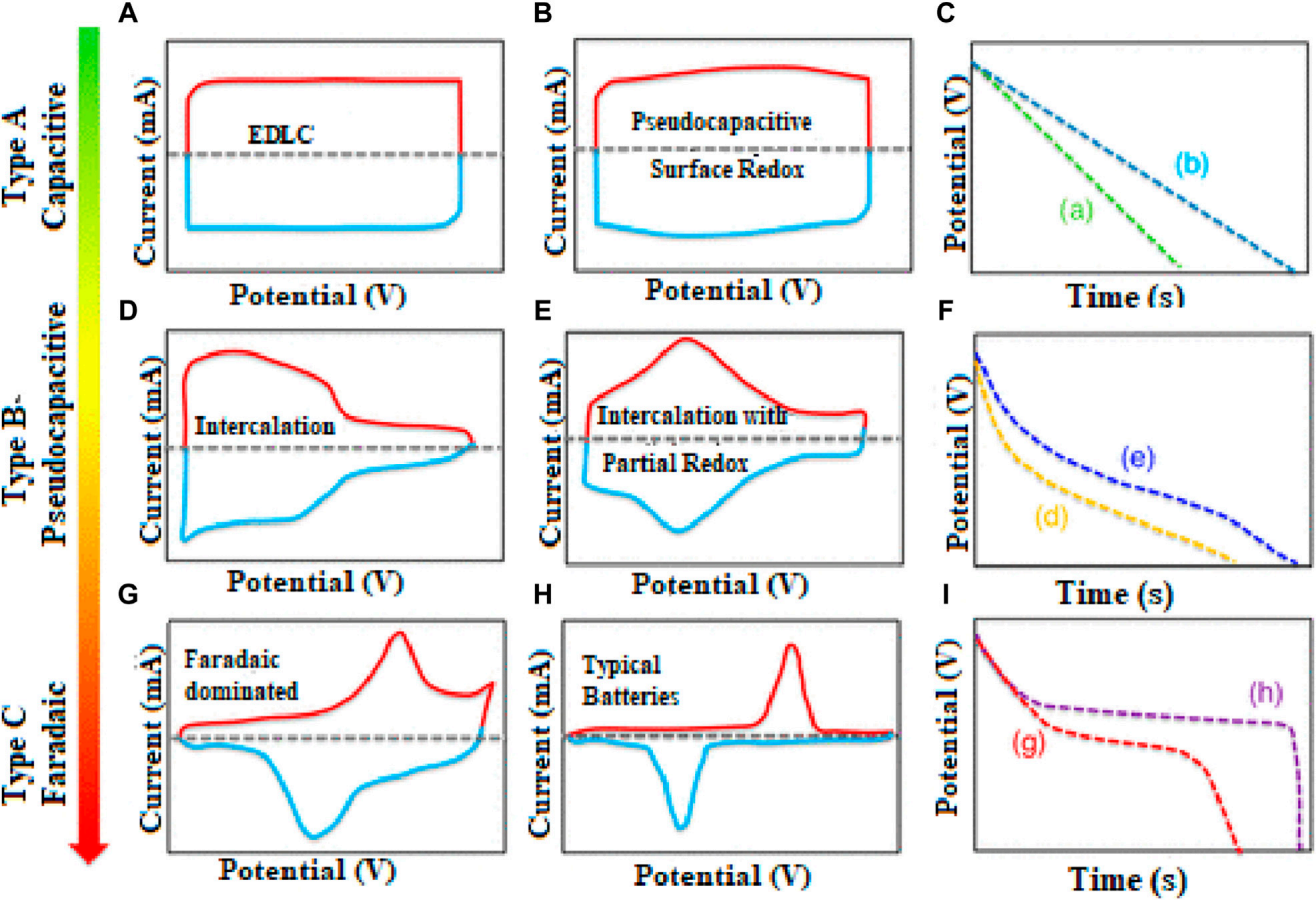
CV plots of **(A)** electrode materials for EDLC, **(B,D, and E)** electrode materials for pseudo-capacitors, **(G)** electrode materials for battery-like, and **(H)** electrode materials for battery and **(C,F, and I)** discharging rate of different materials. Reprinted with permission from Gogotsi and Penner (2018).

On the other hand, electrochemical devices can be differentiated according to the shape of cyclic voltammetry (CV) and galvanometric charge–discharge (GCD) curves. There are several electrochemical characterizations for the materials, but Figure 3 shows the CV and GCD characterization of electrode materials. The CV plots of different SCs and the relation between discharge time and voltage are displayed in Figure 3. Figure 3A shows the CV curve of the EDLC and PCs, which are in rectangular and surface redox forms, respectively, and shows the direct relation between voltage and discharging time. It has been noted that no EC reactions occurred in the charge storage method of EDLC electrode materials, as charge is stored between the surface of electrodes and electrolytes. Figures 3B,D,E show the CV curve of PCs where no phase change occurs in electrode materials. In PCs, chemical processes occur between the electrolytes and the electrode surface, where redox reactions and the interaction of ions are charged in electrode materials. Figures 3G,H display the CV curve of battery-type and pure battery electrode materials, showing a pair of separated redox peaks appearing in both materials and the phase change occurrence in electrode materials (Gogotsi and Penner, 2018; Nan et al., 2019). The comparison of galvanostatic discharging curves of SCs, as shown in Figure 3C, is the discharging curve of EDLCs and PCs, which shows the linear discharging rate. Figure 3F shows the discharging curve of pseudocapacitive materials that indicates a nonlinear discharging rate, and Figure 3I shows the discharging curve of the battery electrode material, which shows that the discharging rate of batteries is slower than that of PCs, hence showing batteries have a high specific capacitance (Nan et al., 2019). It has been noted that the charge storage behavior for the pseudocapacitive electrode material is denoted by specific capacitance, and that for the battery-like electrode material is denoted by specific capacity (Eftekhari and Mohamedi, 2017). Researchers are constantly discovering and improving novel materials and strategies to increase the presentation and energy storage efficiency of SCs.

**FIGURE 4:**
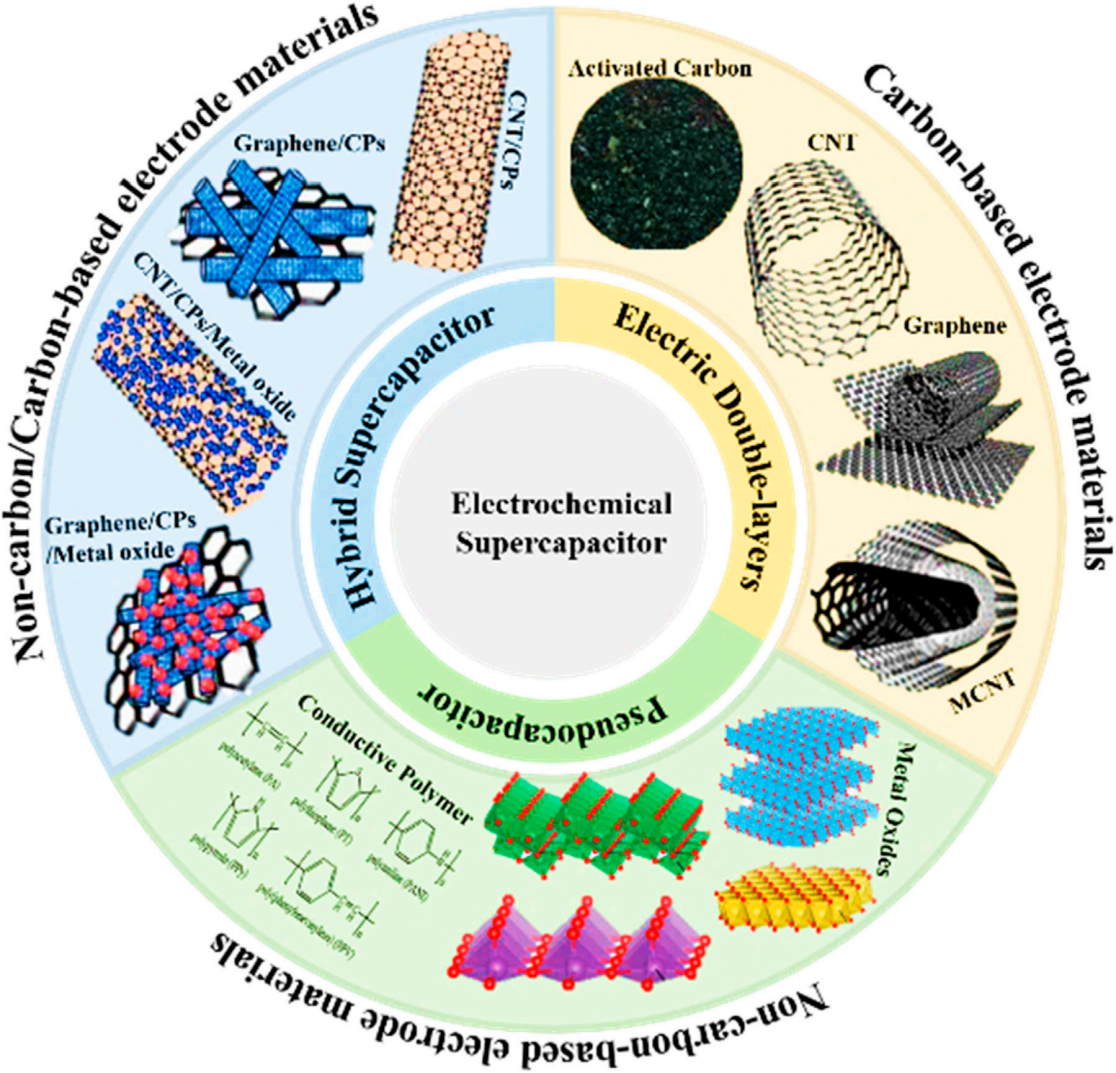
Typical electrode materials for different types of SCs. Reprinted with permission from Hu et al. (2021).

It has been noted that each type of SC has a specific type of electrode material (Hu et al., 2021), as shown in Figure 4. For EDLCs, the electrode materials are CA (carbon aerogels), activated carbon (AC), carbon fibers (CFs), carbon nanotubes (CNTs), graphite, and graphene, which have a good surface area, stability, porous structures, electrical conductivity, functional control, chemical inertness, and a high range of composites with low ED (Wang R. et al., 2021; Ruiz-Montoya et al., 2021). For PCs, the electrode materials are metal sulfide, MOs, metal phosphate, and CP. For ASCs, the efficiency of SC devices can be enhanced by incorporating pseudocapacitive materials into the carbon matrix, and the increase in efficiency is due to the enhancement in Faradic charge storage, coupled with the rapid and fully reversible transfer of electrons and ions, which causes better performance and is more effective. The efficiency of SC devices can also be improved by using hybrid devices based on PC materials and battery-type materials at the anode and cathode (Forouzandeh et al., 2020; Kumar S. et al., 2021; Liang et al., 2021; Qu et al., 2022; Gorsi et al., 2024). Due to the phase transition of an active material and slow reaction kinetics, asymmetric or hybrid cell configurations lower power densities and reduced enduring stability. Thus, improving the ED of SCs while retaining their power and rate cyclability is a critical task for scientists. Most of the past research has been based on carbonaceous materials, and their composites with conducting polymers are employed as electrode materials in SCs because of their high specific capacitance, porous structure, and better stability (Manasa et al., 2022).

Even though there have been numerous studies on MO electrode materials for SC application, it is still critical to investigate recent advances in the safe and sustainable process for synthesizing MO electrode materials, with particular attention given to the design, functionality, and characteristics of SCs.

The novelty of MO electrode materials is primarily due to the presence of synergistic effects in the hybrid materials, high redox activity, excellent conductivity, and chemical stability, which makes them excellent for SC applications (Patil et al., 2022). MOs possess high specific capacitance due to the presence of redox-active sites, allowing them to store a substantial quantity of charge per unit mass (Liang et al., 2021). Additionally, the variable features of MOs can be obtained by the optimization of composition, structure, and morphology, which helps attain particular criteria for SCs in terms of specific capacitance (Cs), cyclic stability, and ability to store charge for a long time (Ansari et al., 2022). This tunability can maximize charge storage, while also improving ion transport and kinetics, which provides a level of precision that other types of electrode materials may struggle to achieve. Further investigation and advancement in this domain might potentially enhance the efficiency and suitability of MO-based supercapacitors across a range of energy storage applications.

In this review, we generally focus on the pseudo-capacitors SCs, which mainly contain MOs and their composites with conducting polymer electrode materials. Additionally, we also explain some composites of MOs with carbon-based materials for SC applications.

## 2 Synthesis methods and morphological study of MO materials

MO-based electrode materials possess high C_s_, have a higher energy density than carbonaceous materials, and are more stable than conductive polymers (CPs) (Liang et al., 2021; Tadesse et al., 2024). Currently, most researchers are working on different types of MOs and their composites with C-based and non-C-based materials. The most important and abundant metal oxides in nature are Co_2_O_3_, MnO_2_, Co_3_O_4_/NiO, and NiO, which are considered good electrode materials for SCs (Xu et al., 2007; Li et al., 2011; Fan et al., 2012; Shariq et al., 2023; Sohail et al., 2024). Several composites of MOs with carbonaceous materials, such as reduced graphene oxide (rGO), graphene oxide (GO), and graphite (G), have been prepared using various methods of preparation with varying morphologies. Examples include CuO/rGO (Bu and Huang, 2017), NiO/rGO (Kumar et al., 2020), MnO_2_/rGO (Liu et al., 2017), TiO_2_/rGO (Ramadoss et al., 2013), and rGO/Co_3_O_4_/CoO (Kumar R. et al., 2021), as well as NiO/rGO (Li et al., 2021). Additionally, nanocomposites of NiO/ZnO (Anandhi et al., 2019) and ZnCo_2_O_4_ (Chen et al., 2020b), composites of NiO and CoO (Li et al., 2020), ternary MO like Zn-Ni-Co (Wu et al., 2015) and CeO_2_/ZnO/ZnWO_4_ (Khawar et al., 2022), and composites of Zn-Co-Mo with rGO (Liu et al., 2023) have been published, which were prepared by different techniques and offer excellent electrochemical properties. It has been noted that synthesis methods, crystal size/shape, and morphology of the materials play an important role in the EC properties of the material. However, the morphology and crystal size/shape of the materials can be adjusted with the help of synthesis methods.

### 2.1 Synthesis of MO materials

**FIGURE 5:**
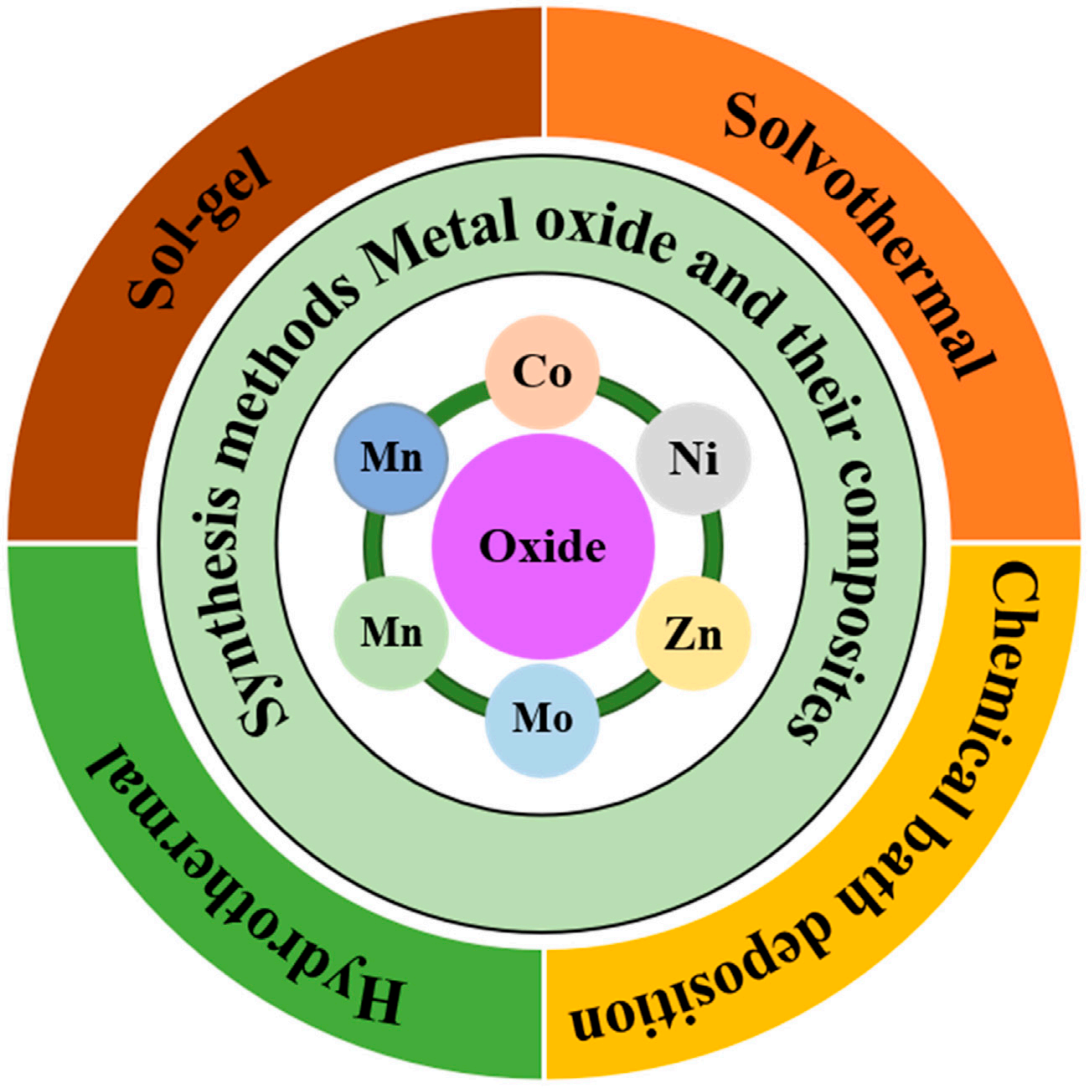
Synthesis methods of electrode (MO) for supercapacitor. Reprinted with permission from Kumar A. et al. (2022).

MOs and their composite-based electrode materials for SCs can be synthesized using various techniques. It has been considered that for high-performance SCs, a prepared electrode material must have the appropriate structure, size, and morphology. In this regard, numerous synthesis methods have been used by researchers to prepare the different nanostructures of composites with different dimensions. The choice of the synthesis method affects the structure, morphology, surface area, and EC properties and ultimately influences the performance of SCs. Some synthesis methods for MOs and their composites are shown in Figure 5.

To synthesize MOs and their composite materials, a one-step process called the hydrothermal method can be used. The material synthesis by the hydrothermal method is attributed to high temperature and high pressure, which generate synergistic effects (Lu et al., 2017). There are various ways in which a material could be modified, such as by changing the precursors, the ratios of the reactants, and the temperature of the heating process. The one-step process is an effective method to activate morphology and change particle size; it is simple, low in cost, environmentally friendly, and appropriate for large-scale production.

In the solvothermal process, a non-aqueous solvent (organic solvent) is used as a reaction medium under a wide range of temperatures, preventing the formation of agglomerations and controlling the morphology and structure of the products in the reaction. Due to the morphological changes in nanostructured materials, there is a reduction in diffusion length for ions, which facilitates the facile movement of ions and electrons in the electrolyte and enhances the EC performance of the material (Shi J. et al., 2020). The electrodeposition method deposits metallic coatings on substrates by reducing aqueous or organic precursor solutions with a cathodic current based on cathodic reduction action. This process extensively prepares nanostructured electrode materials based on MOs on varied substrates to be uniformly deposited over the electrodes. This process has some advantages, such as occurring at room temperature, being cost-effective, simple, and also good for controlling the thickness of the electrode, structure, and morphology by changing the deposition time, precursor solution, and current (Lu et al., 2017). The sol–gel process takes place at low temperatures and is cost-effective. In this method, sols and gels are formed by hydrolysis and condensation (condensation is affected by some parameters, such as the ratio of water and alkoxide, temperature, pH, solvent, and employed catalyst) (Asghar et al., 2022a). The co-precipitation method has several steps (nucleation, growth, coarsening, and aggregation). The first step involves the formation of small particles, and the second step involves a disturbance in the size, properties, and morphology. The co-precipitation method is very simple, fast, and well-organized (Zhang G. et al., 2017). Different types of inorganic nanomaterials can be prepared by electrospinning, including nanowires and nanotubes that can be grown without disturbing the crystal growth process. The electrospinning method is used for forming fibrous nanostructures. The liquid precursors are ejected from the tube during spinning, where the electric field gains the spinning force. On the other hand, a number of other preparation routes, including pulsed laser ablation (PLA), successive ionic layer adsorption and reaction (SILAR), microwave irradiation (MWI), and combustion-driven processes, have all been used to prepare the metal oxides and their composite-based electrode materials (Bai et al., 2020; Park et al., 2020; Raut et al., 2021; Samal et al., 2021). Among these methods (PLA, SILAR, and MWI), the MWI method is useful for the synthesis of MOs and their composite-based electrodes materials for SC applications, which yields a variety of multimodal morphology and crystal architectures of electrode materials. However, the MWI synthesis method has some limitations, such as equipment availability, cost-effectiveness, and restricted large-scale production. It has been noted that for implementing high-performance ESDs, various parameters (temperature, pressure, time, ratio of reactants, etc.) are related to the above-discussed synthesis process that can be adjusted to attain the optimal materials for various applications. The crystallite size and morphology of the prepared material play an important role in the EC performance of the electrode materials.

### 2.2 Basic characterization techniques

X-ray diffraction (XRD), scanning electron microscopy (SEM), and transmission electron microscopy (TEM) are the basic techniques that contribute to understanding the structure–property relations of MO electrode materials. XRD is useful for finding crystalline structures in MO electrode materials, which facilitates understanding of how structural characteristics influence electrochemical performance. It has been noted that changes in the crystal structure, as well as the presence of contaminants, can have an impact on electrode material functionality. SEM evaluates the properties of electrode materials including particle size, shape, and dispersion of the surface in metal oxide-based electrode materials. Studying the shape of the surface is important for optimizing factors such as reactive surface area, which have a direct impact on the electrode’s functionality in terms of storage of charge ability, rate capability, and cycle stability. TEM enables observing the nanostructure, crystallographic flaws, edges of grains, and interfaces of the materials used for electrodes. Information about the nanoarchitecture is important for understanding some phenomena such as ion dispersion, transfer of electrons, and phase transitions in electrochemical reactions.

### 2.3 Morphological study of MOs and their composites

**FIGURE 6:**
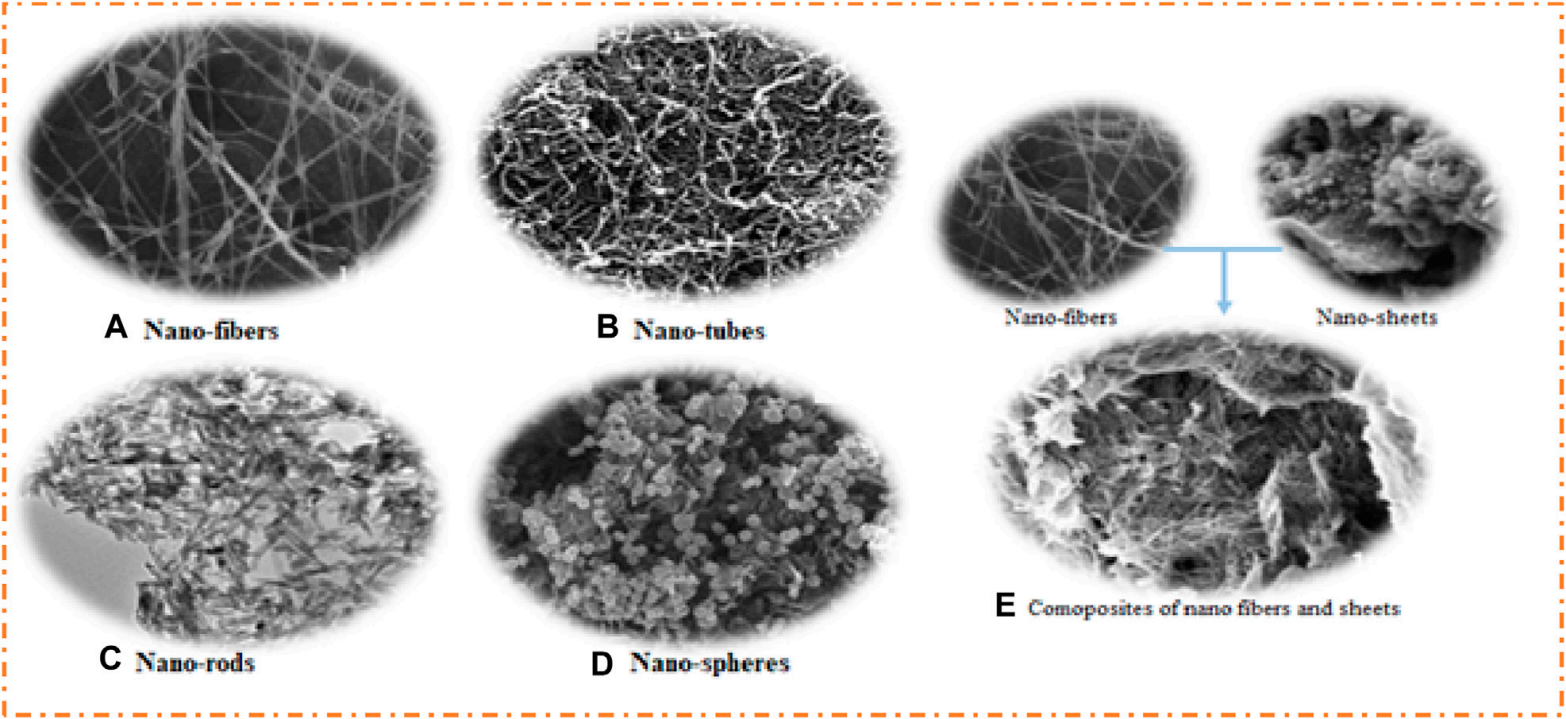
SEM images of **(A)** NiFO_f_, **(B)** NiFO_t_, **(C)** NiFO_r_, **(D)** NiFO_s_,and **(E)** composite of AC and NiFO_f_. Reprinted with permission from Toghan et al. (2022).

The physical properties of materials depend on production routes, surface-to-volume ratio, and grain boundaries that affect the electrical, optical, mechanical, magnetic, and electrochemical properties of the materials (Jeevanandam et al., 2018; Khan I. et al., 2019; Kanwal et al., 2023). As a result, the conductivity at the grain boundary and other surface characteristics like roughness play an important role in the energy storage of materials. Additionally, it has been noted that nanoparticles have numerous applications in electronics, sensors, batteries, fuel cells, and supercapacitors due to their outstanding surface-to-volume ratio and conductivity. Figure 6 shows the morphological effects on the electrochemical properties of NiFe_2_O_4_ composites, which have been prepared using different fabrication methods like sol–gel, hydrothermal, green, and electrospinning. It has been observed that the morphology of NiFe_2_O_4_ has changed by use of the changing preparation method. The main morphology of NiFe_2_O_4_ obtained includes nanofibers (NiFO_f_) by electrospinning, nanotubes (NiFO_t_) by hydrothermal, NiFe_2_O_4_, nanorods (NiFO_r_) by sol–gel, and nanospheres (NiFO_s_) using the green process (Hamza et al., 2022; Toghan et al., 2022). Figures 6A–D show the SEM images of NiFO_f_, NiFO_t_, NiFO_r_, and NiFO_s_, respectively. These images demonstrate the approximate calculations of diameter/length: 55 nm/few millimeters for the nanofiber structure, 9 nm/230 nm for the nanotube structure, 30 nm/150 nm for the nanorod structure, and 35 nm diameter for the spherical structure. The composite of NiFO_f_ and AC sheets demonstrates a mixed morphology with a significant decrease in fiber lengths. Additionally, it was noted that the AC sheets covered the NiFO_f_ fibers. The impact of morphological structure on electrochemical characteristics and their utilization in energy storage was examined, which shows that nanofibers exhibit an excellent surface area, a higher specific capacity of 1,260 F g^−1^, and a smaller particle size, as compared to other structures. The specific capacitance (1,283, 1,140, 680, 550, and 384 F/g) at a scan rate of 5 mV/s, specific capacitance (1,260, 1,130, 660, 520, and 340 F/g) at a current density of 1 A/g, particle sizes (15, 20, 33, 41, and 50 nm), and surface areas (2,570, 360, 115, 82, and 54 m^2^/g) of electrode materials are in the order of AAC@NiFO_f_, NiFO_f_, NiFO_t_, NiFO_r_, and NiFO_s_, which have the same trend as of particle size (decreasing) and surface area (increasing) of the electrode materials. From these results, it can be inferred that the preparation technique has a significant impact on crystallite size, material shape, surface area, and specific capacitance.

**SCHEME 1:**
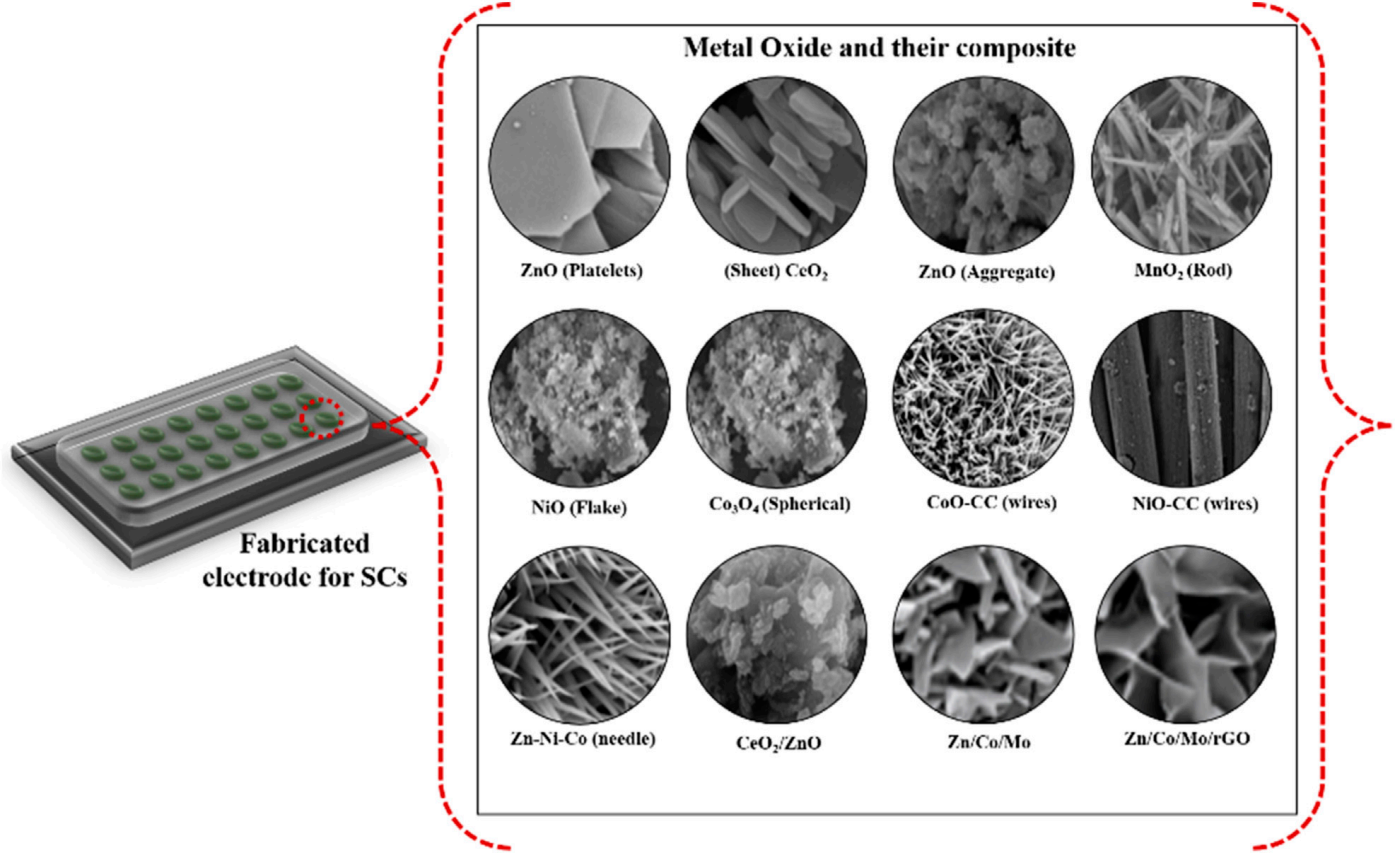
Morphology of different metal oxides and their composites. Reprinted with permission from Xu et al. (2007); Li et al. (2011); Fan et al. (2012); Wu et al. (2015); Li et al. (2020); Khawar et al. (2022); Liu et al. (2023).

In this review, we will mainly focus on the MOs and their composite-based electrode material, and the morphology of different MOs and their composites is shown in Scheme 1. It is important to note that the performance and characteristics of oxide and their composite-based SCs can vary depending on the morphology of materials, the specific metal used, the electrode structure, and the overall device configuration (Li et al., 2011; Wu et al., 2015; Chen et al., 2020b; Li et al., 2020; Li et al., 2021; Liu et al., 2023). Ongoing studies and advancements aim to optimize these SCs further for improved energy storage capacities and broader applications in everyday life.

In summary, the synthesis methods, morphology, and crystal size/shape of the materials play an important role in the electrochemical properties of the electrode materials (Wu et al., 2015; Chen et al., 2020b; Li et al., 2020; Li et al., 2021; Liu et al., 2023). Researchers can modify and control the morphology/crystal size/shape of the MO electrode materials throughout synthesis and also can adjust the capabilities of MO electrode materials for specific purposes and applications. With the help of various synthesis methods, like hydrothermal synthesis, sol–gel, co-precipitation, solvothermal, electro-deposition, and template-assisted methods, we may achieve the desired morphologies.

In all synthesis methods, the one-step hydrothermal procedure is an excellent method to enhance the morphology and modify particle size/shape. Additionally, the hydrothermal method is effortless, cost-effective, ecologically beneficial, and suitable for large-scale production. On the other hand, the MWI approach has limitations in terms of equipment availability, cost-effectiveness, and restricted large-scale manufacturing, but it is also an efficient way for producing MOs and composite electrode materials for SC applications, which yields a variety of multimodal morphology and crystal architectures of electrode materials. Additionally, the area of the surface and conduction of the materials, ion transport, durability of structures, and Faradaic responses of the metal oxide electrode materials also have a considerable impact on their electrochemical properties (Tue et al., 2023).

Nanostructured substances provide a smaller path of diffusion; increase the integrity of the structure, resulting in a quicker rate of charge as well as discharge; and reduce the rate of disintegration with frequent cycling. The significant area of the surface of electrode materials provides higher-activity sites for electrochemical processes. Research suggests that electrode materials that have small internal resistances, as well as elevated transfer rates of electrons, are extremely conductive (Gao and Zhao, 2022).

## 3 MOs and their composite-based electrode material

Numerous metal oxides such as nickel oxide (NiO), ruthenium oxide (RuO_2_), cobalt oxide (Co_3_O_4_), and manganese oxide (MnO_2_) are normally used as electrode materials in MO-based SCs. These MO-based materials offer high specific capacitance and good cycling stability, which permits oxide-based SCs to survive several cycles of charging and discharging without degradation. This property is important for the long-term reliability and durability of the device (Liang et al., 2021). Metal oxide-based SCs store energy through a pseudo-capacitance mechanism where the MO electrode is in contact with the electrolyte. Reversible redox reactions occur at the electrode and electrolyte interface, further storing charge beyond the electrostatic double-layer capacitance. MO-based SCs have the potential to attain higher energy densities as contrasting to the EDLCs due to their additional pseudo-capacitance, which makes them smart for applications that need high power and energy storage capabilities.

This section provides a brief overview of single MOs, bi-MOs, and tri-MOs, as well as their composites with carbon-based electrode materials for SC applications.

**FIGURE 7:**
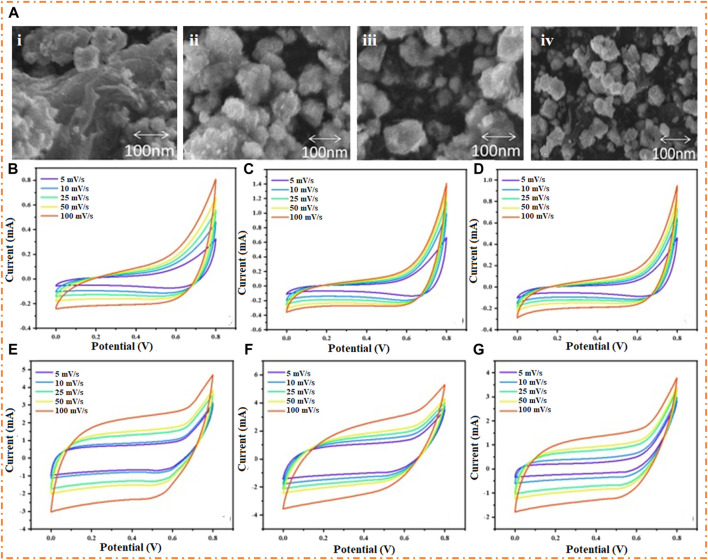
**(A)** SEM image of Co_3_O_4_ (i) at low temperatures, (ii) 200°C, (iii) 250°C, and (iv) 300°C. Cyclic voltammetry of Co_3_O_4_ at **(B)** 200°C, **(C)** 250°C, and **(D)** 300°C under pH = 7, 6, and 11 **(E–G)**. Reprinted with permission from Jennifer et al. (2022).

Jennifer et al. (2022) prepared the Co_3_O_4_ nanomaterial using the hydrothermal method and studied the structural, morphological, functional, and electrochemical properties at different temperatures. It has been shown that at 250°C, the material Co_3_O_4_ has a 215 F/g specific capacitance with a low series resistance of 2.6 Ω and a large area under the curve, proving it to be a good material for SCs. The material’s morphology varies with changing temperature, as shown in Figure 7A(i-iv). At 300°C, the material attains good morphology, and the observed grain size of the cobalt oxide was 40 nm, which may be due to the aggregation of the Co_3_O_4_ nanosphere. It has been noted that the electrochemical properties can be changed by varying the doping concentration or temperature. The bunch of nanospheres was responsible for enhancing the diffusion rate but affected the charge transfer, which caused a reduction in specific capacitance. The varying size of the Co_3_O_4_ nanosphere also affects the ED and PD. The ED was 68.69 Wh/kg at a PD of 0.5 kW/kg, and a PD of 2.5 kW/kg was gained at an ED of 21 Wh/kg in the presence of a 1 M solution of the Na_2_CO_3_ electrolyte. Figures 7B–D show the CV curves of Co_3_O_4_ at different temperatures (200°C, 250°C, and 300°C) with the Na_2_CO_3_ electrolyte having a pH of 7. The highest specific capacitance of the prepared material (215 F/g) is observed at 250°C, and large areas under the curve are kept at the same temperature with different scan rates (5 to 100 mV/s). This showed that a temperature of 250°C is considered good for EC applications. Figures 7E–G show the CV curves at different temperatures (200°C, 250°C, and 300°C) with the 2 M electrolyte (Na_2_CO_3_) having a pH of 11. This result also showed that a temperature of 250°C is good for EC properties. Vinodh et al. (2022) synthesized the NiO NPs by the hydrothermal method; annealed the material at 300°C, 500°C, and 700°C; and observed the morphology and EC behavior of the material in the presence of a 2 M KOH electrolyte. At 300°C, the prepared material NiO showed a good capacitance of 569 F/g at 0.5 A/g. The fascinating morphology of NiO explains a critical part of its ability to carry ions, confine electrons, and allow ions to pass to provide EC reactions with simple ion transport. The ASC was fabricated with the help of NiO as the positive electrode and AC as the negative electrode, gained good PD 800 at 52.4 Wh/kg ED, and had good cyclic stability.

**FIGURE 8:**
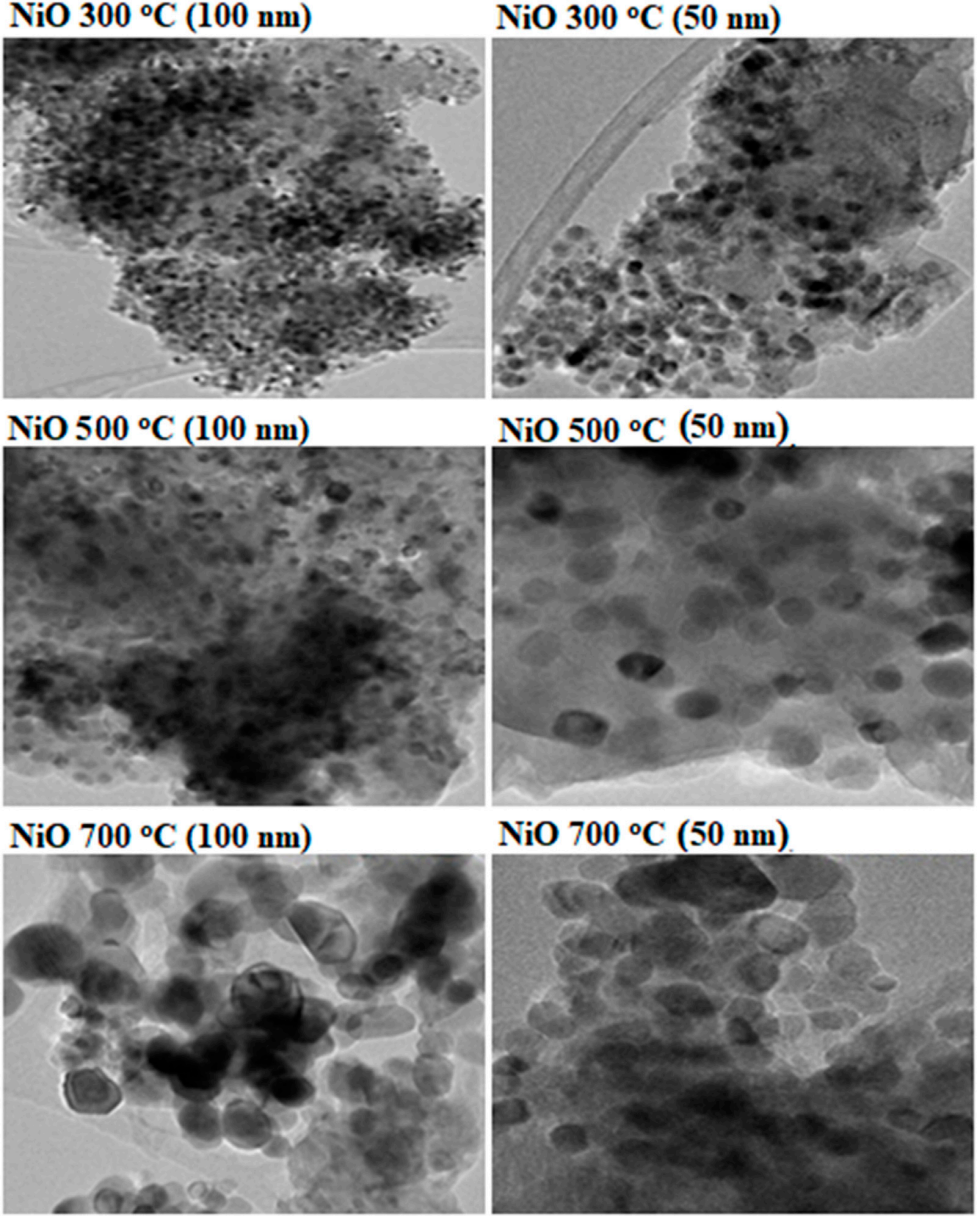
TEM images of NiO at various temperatures (300°C, 500°C, and 700°C). Reprinted with permission from Vinodh et al. (2022).

The flake-like TEM images of NiO at 300 °C, 500 °C, and 700 °C are shown in Figure 8. It can be seen that at 300°C, an excellent morphology was observed. At 300°C, a well-ordered channel with small pores was detected, whereas the particles of NiO were closer to each other, emerging a fine track within the flake-like structure. It has been noted that increasing temperature causes NiO’s growth with crystal size enhancement and an uneven surface, which concludes that large particles were observed within the flake structure (Li et al., 2011).

**FIGURE 9:**
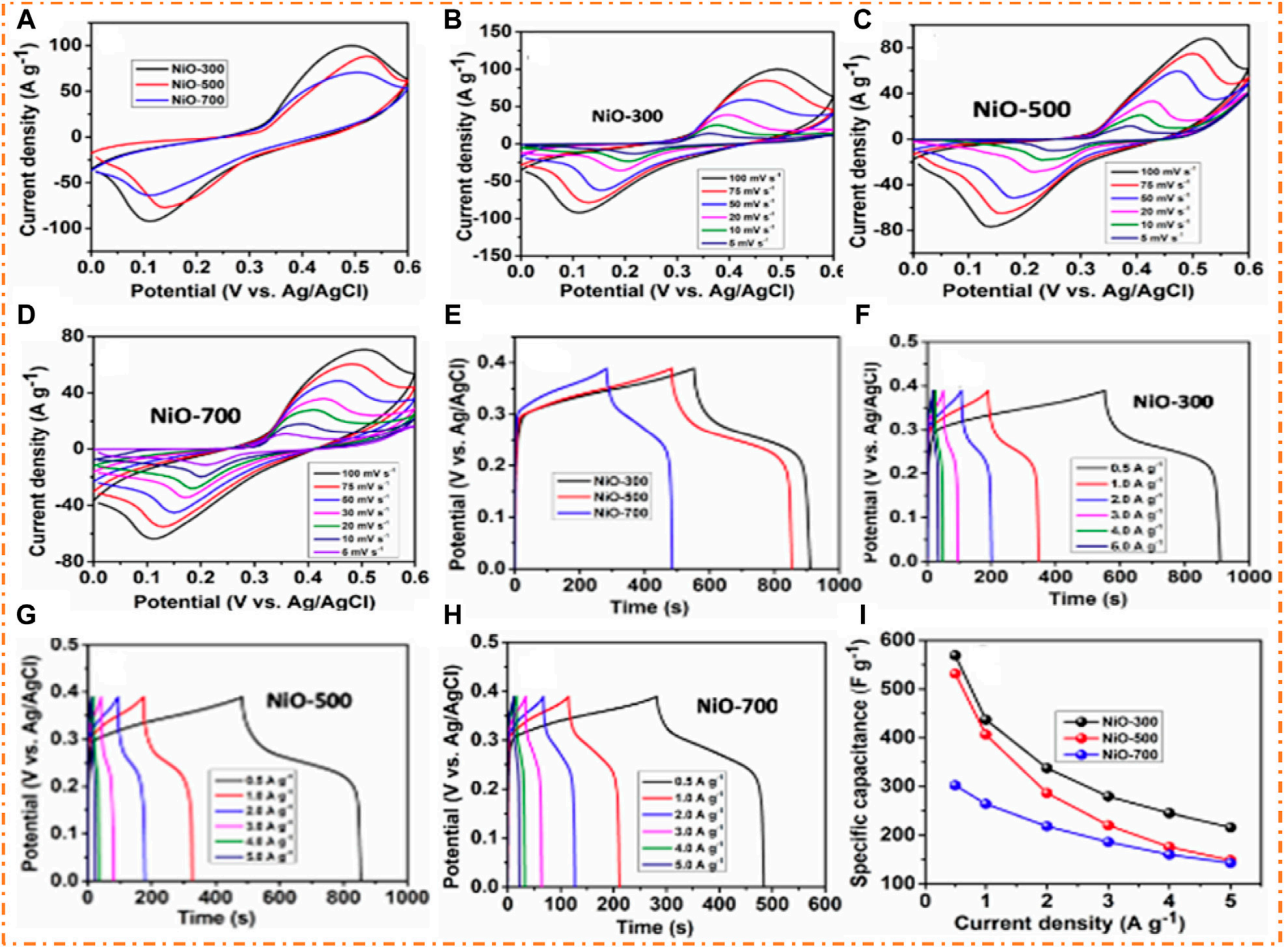
**(A)** CV curve of three NiO electrodes, **(B–D)** CV curve of NiO at 300°C, 500°C, and 700°C and GCD curve of **(E)** three NiO electrodes. **(F–H)** GCD curve of NiO at 300°C, 500°C, and 700°C, and **(I)** specific capacitances vs current densities. Reprinted with permission from Vinodh et al. (2022).

The CV curves of NiO of three samples (300°C, 500°C, and 700°C) with various scan rates (5, 10, 20, 50, 75, and 100 mV/s) are shown in Figure 9. During several scan rates, it has been noted that NiO oxidizes in the KOH electrolyte, which showed that a redox reaction (Ni^+2^ to Ni^+3^) takes place at the surface of the electrode, which causes enhancement in the capacitance. Observing the CV curve, which is the same as the original redox peak, confirmed pseudo-capacitance performance. Figure 9A shows the comparison of CV curves of NiO observed at different temperatures. Figure 9B ensures that the current response at a temperature of 300°C, compared to Figures 9C,D, exhibits a high surface area with a porous structure and provides an easier path for the diffusion of ions. The GCD curves of NiO at several temperatures (300°C, 500°C, and 700°C) by employing three electrode systems with different CD 0.5–20 A/g and also at a potential window of 0–0.40 V are also shown in Figure 9. Figure 9E shows the combined picture of the GCD of three electrodes of NiO at different temperatures. Figures 9G,H show that the GCD curves at 500°C and 700°C exhibit lower values of charge kinetics compared to those at 300°C, which exhibit a fast charge and discharge mechanism, as clearly depicted in Figure 9F. The GCD plots are nearly symmetric, and the response of the charge/discharge plateau is a clue to the redox route of NiO. The gravimetric capacitances of NiO are predictable from the discharge shapes, as shown in Figure 9I, which shows that at 300°C, the electrode has a higher discharging time, which confirms the specific capacitance of 569 F/g. It is also concluded that with increasing temperature, the value of Sc decreases, which limits kinetics by electrolyte KOH and ion diffusion into the materials (Vinodh et al., 2022). Sethi et al. (2021) fabricated the electrode material of NiO by employing the solvothermal method and detected the electrochemical behavior of the material in a 2 M KOH electrolyte. The electrode material of NiO exhibits 305 F/g specific capacitance and a high PD of 8 kW/kg due to the higher conductivity. Li et al. (2021) prepared NiO and rGO composites using the hydrothermal method and studied the EC behavior of electrode materials. NiO/rGO exhibited good specific capacitances of 435 F/g and 400 W/kg PD at 77 Wh/kg ED.

**FIGURE 10:**
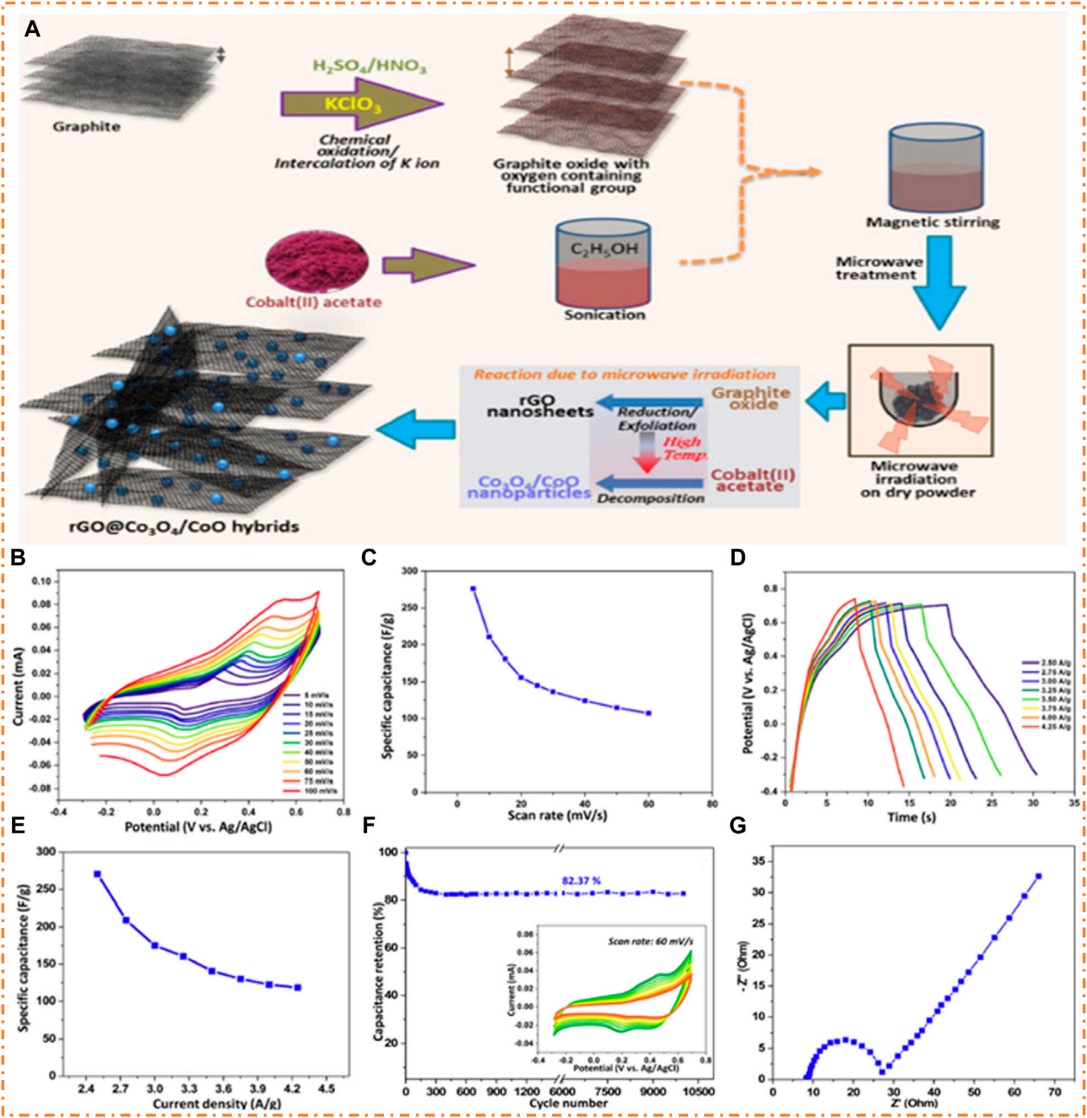
**(A)** Synthesis method of the rGO@Co_3_O_4_/CoO composite, **(B)** CV plots, **(C)** SC at various SR, **(D)** GCD plots, **(E)** SC at various current density (CD), **(F)** capacitance retention, and **(G)** Nyquist plots of rGO@Co_3_O_4_/CoO hybrids. Reprinted with permission from Kumar R. et al. (2021).

Kumar R. et al. (2021) found a simple and fast way of synthesizing rGO@Co_3_O_4_/CoO composites for advanced SCs by using microwave technology within a short period of only 1.5 min. The hybrid composites exhibited good EC properties, such as Cs and cycling stability, which gave good results compared to individual MOs. In the future, graphene derivatives with other MOs can be synthesized for energy-related applications using this microwave-based synthetic approach. The mechanism for fabricating the rGO@Co_3_O_4_/CoO composite was the microwave-supported reduction and exfoliation method. In this process, the GO is reduced to rGO by applying a high temperature using a microwave and a porous nanosheet of rGO is produced with emissions of CO and CO_2_. Oxygen functionalities are removed when graphite oxide is exposed to high temperatures, and gases expand (Pei and Cheng, 2012). In this synthesis, the possible condition for preparing rGO@Co_3_O_4_/CoO is the presence of GO with cobalt acetate. The overall mechanism for the formation of composites rGO@Co_3_O_4_/CoO is shown in Figure 10A. The CV plots of rGO@Co_3_O_4_/CoO are shown in Figure 10B, which is not in a rectangular form that denotes PC and EDLC performance. Two redox peaks also are observed for SC electrode materials, indicating the emergence of pseudo-capacitance from the changeable redox reaction of Co_3_O_4_/CoO NPs in hybrid materials. From the CV plots, the prepared material exhibited a maximum specific capacitance of 276 F/g at a scan rate of 60 mV/s, as shown in Figure 10C. The GCD plots of rGO@Co_3_O_4_/CoO are demonstrated in Figure 10D, which clear the irregular behavior of the charging and discharging graph due to the occurrence of the redox reaction of the NPs of rGO@Co_3_O_4_/CoO. Moreover, from GCD plots, the maximum specific capacitance of 271 F/g was measured at a CD of 4.25 A/g, as shown in Figure 10E. The cyclic stability of electrode rGO@Co_3_O_4_/CoO is shown in Figure 10F. It was found that after 10,000 cycles, the capacitance retention was 82.4%. The gained EC values of rGO@Co_3_O_4_/CoO explain good behavior as compared to formerly fabricated electrode materials. The electrical behavior of rGO@Co_3_O_4_/CoO measured by EIS and Nyquist plots of the electrode is shown in Figure 10G. The Nyquist plot characterizes the frequency responses of the electrolyte and electrode material. At low frequencies, the straight line can be attributed to the diffusion of ions through the electrode material (Kumar et al., 2017). Moreover, the reasonable resistance of the solution shows the compressible and conductive nature of the electrode material.

On the other hand, bi-metallic and tri-metallic oxides have higher ED than single MO because of their better redox reactions, high electrical conductivity, and high storage capacity. Anandhi et al. (2019) synthesized the electrode material using the sol–gel method and increased the EC behaviors of NiO and ZnO nanocomposites for SCs. The prepared material has a good Cs of 469 F/g and an excellent PD of 1458 W/kg at an ED of 91 Wh/kg.

**FIGURE 11:**
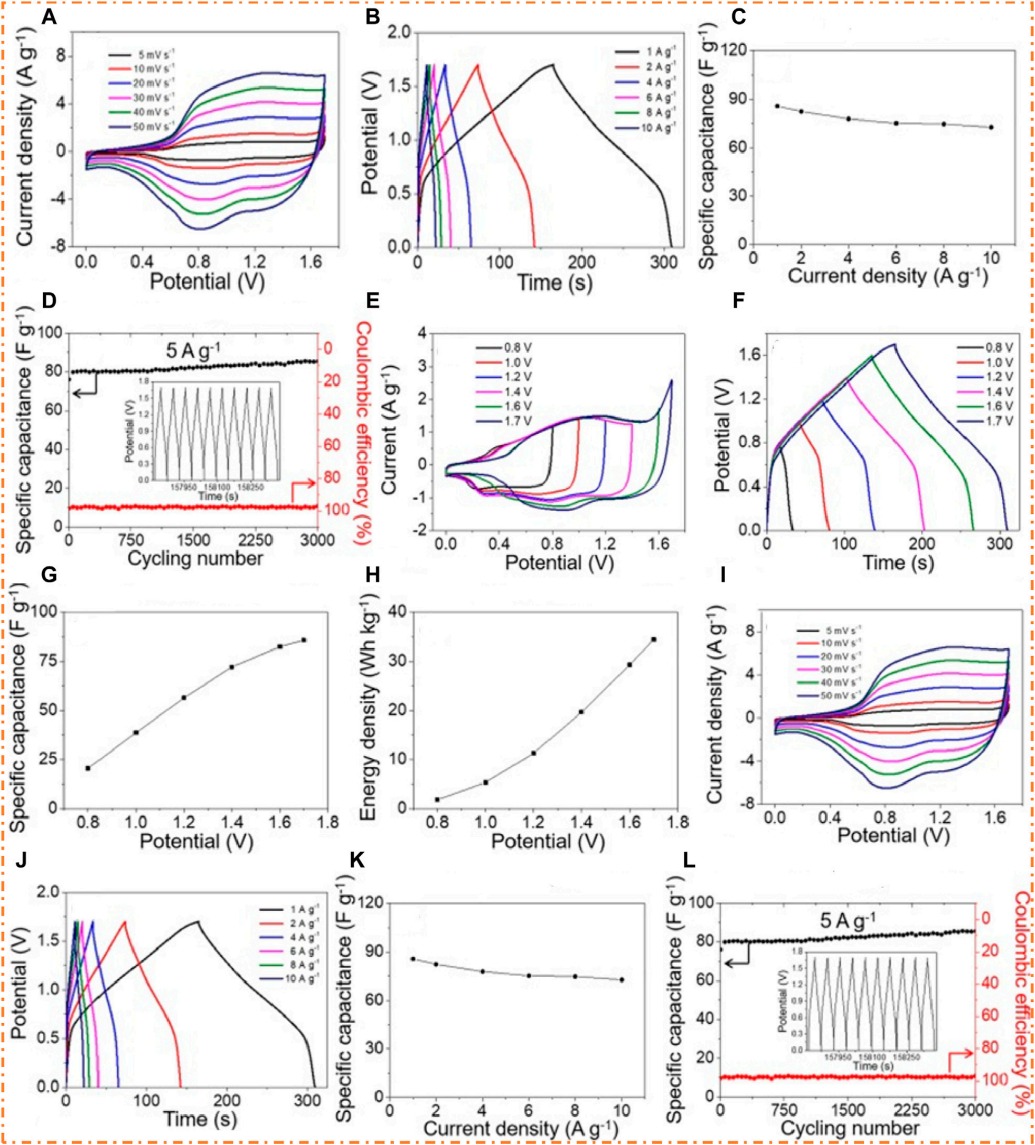
EC tests of ZnCo_2_O_4_ cubes: **(A)** graph of CV, **(B)** graph of GCD, **(C)** graph of SC vs. CD, and **(D)** cycling presentation and Coulombic efficiency; EC presentation of the ZnCo_2_O_4_-AC ASSC device, **(E)** graph of CV (without scan rate), **(F)** graph of GCD (without current density), **(G)** specific capacitance vs. potential window, and **(H)** energy density vs. potential window; graph of ZnCo_2_O_4_-AC ASSC device **(I)** CV at different scan rates, **(J)** GCD at different current densities, **(K)** graph of SC vs. CD, and **(L)** cycling stability and Coulombic efficiency with last 10 cycles of GCD in the inset. Reprinted with permission from Chen et al. (2020b).

Chen et al. (2020b) prepared ZnCo_2_O_4_ cubes with an average size of 8 nm and a surface area of 84 m^2^/g. They found good values of EC properties, such as a specific capacitance of 804 F/g and stability of 79% after a 3 K cycle. Furthermore, they fabricated the ZnCo_2_O_4_-AC device, which shows an ED of 34 Wh/kg at a PD of 860 W/kg and a decent retention value of 112% after a 3 K cycle. The CV graph is shown in Figure 11A, which denotes that the ZnCo_2_O_4_ electrode exhibits pseudocapacitive behavior. The peaks confirmed that the increase in the scan rate caused the shifting of the oxidation peak from lower voltage to high voltage, and the reduction peak moved in the reverse direction. It is believed that the oxidation peak fluctuates in a positive potential direction, as the reduction peak fluctuates in a negative direction, resulting mainly from the polarization effect on the oxidation peaks (Asghar et al., 2022b). The GCD plots of ZnCo_2_O_4_ are displayed in Figure 11B, which has a plateau portion that confirms the pseudocapacitive nature of the ZnCo_2_O_4_ electrode. ZnCo_2_O_4_ had a Cs of 804 F/g as measured by GCD, as shown in Figure 11C. It has been noted that the high-current-density electrolyte ions may insert and deinstall, and mechanical stress is caused, decreasing specific capacitance. The cycling presentation was measured by GCD for 3 K cycles. It is clearly shown in Figure 11D that for the first 600 cycles, the Cs decreased (683–540 F/g) and remained constant for the next 2,400 cycles. The cause of a decrease in Cs may be attributed to the destruction of ZnCo_2_O_4_ cubes during the preparation of the working electrode or due to the collapsing of cubes (Sahoo and Shim, 2017; Gao et al., 2018). There was a 79% retention in capacitance over 3 K cycles, which suggests that porous ZnCo_2_O_4_ has good electrochemical cycling durability over an extended lifetime.

Figure 11E displays the fabricated device’s CV (without scan rate) graph, which clearly shows that there was no polarization in the ASSC device with the increasing potential window. Figure 11F displays the graph of the GCD of the fabricated device, which clearly shows that no polarization occurs in the ASSC device with the increasing potential window. It is observed in Figures 11G,H that there might be a possible increase in the specific capacitance (21–86 F/g) and the ED (1.8–34 Wh/kg) of the ASC device due to its wider potential window (0.8–1.7 V). It is important to emphasize that a suitable PW must estimate the electrochemical presentation of the ZnCo_2_O_4_–AC ASSC device. Figure 11I shows the graph of the CV of the fabricated ASSC device at various scan rates (SR) among a potential window. As can be seen from the partial rectangular CV curves detected for each scan rate, it is evident that the EDLC and pseudo-capacitance influence capacitance. Figure 11J displays the graph of the GCD of the fabricated ASSC device (ZnCo_2_O_4_-AC) at different CDs among a potential window. The supreme value of the specific capacitance was 86 F/g at CD, as shown in Figure 11K. It has been found that when the fabricated device is tested with increased CD, it displays decent rate competence, as shown by 85% capacitance retention with increasing CD. The cycling performance of the device was assessed by repeating the charging and discharging processes on the same CD over cyclic stability with different potential windows. A particularly remarkable outcome of the experiment was that after 3 K cycles, specific capacitance remained at about 112% of its initial value, and the Coulombic efficiency remained pretty much unchanged from 100% throughout, as shown in Figure 11L. In the inset of Figure 11L, we can see the graph of the GCD of the last 10 cycles, and the charging curves are proportional to the discharging curves; therefore, there is no potential drop in the charging curve. As a consequence of all the above studies, the ZnCo_2_O_4_-AC ASSC has been found to have excellent EC stability.

**FIGURE 12:**
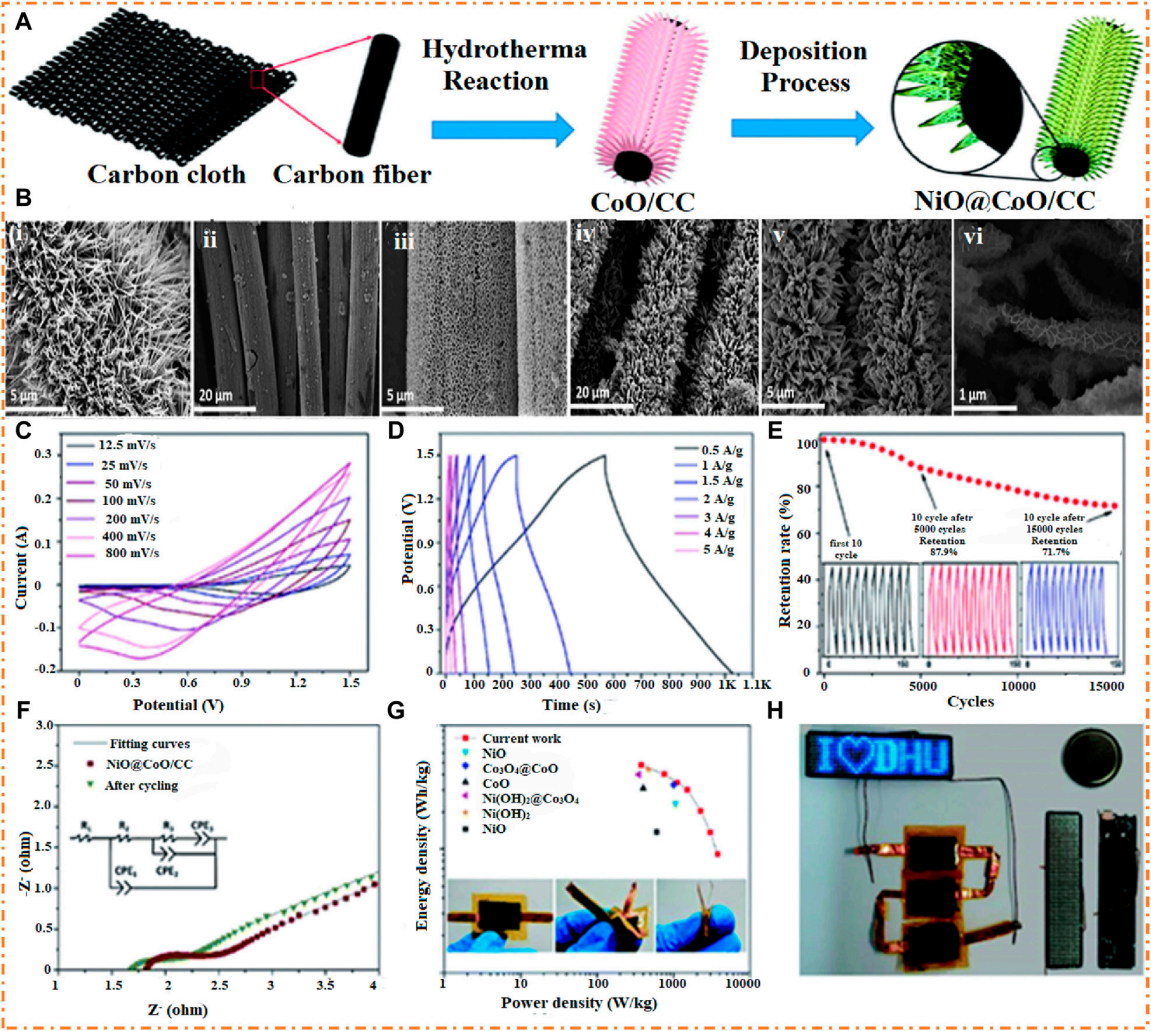
**(A)** Representation of the preparation of NiO-CoO/CC. SEM images of **(B)**-i CoO-CC, **(B)**-ii–iii NiO-CC, **(B)** iv–vi composites NiO-CoO-CC at several magnifications and EC performance of NiO-CoO/CC, **(C)** graph of CV, **(D)** graph of GCD, **(E)** performance of the SC device, **(F)** Nyquist plots, **(G)** Ragone plot, and **(H)** ASSCs powering the programmable LED. Reprinted with permission from Li et al. (2020).

Li et al. (2020) prepared the NiO-CoO composite on carbon cloth (CC) and employed it as an electrode material for SC applications. The specific capacitance of the NiO-CoO electrode was 1,024 F/g, and the retention was 81% after 5 K cycles. The fabricated core-shell-like ASSC electrode has an ED of 40 Wh/kg at a PD of 750 W/kg with a good retention of 72% after 15 K cycles. Figure 12A shows the NiO-CoO composite synthesis on the CC. In this procedure, the CC is used as a substrate, and then CoO is added to the CC via a hydrothermal process. After that, NiO is grown on CoO-CC by utilizing a bath deposition method. At the end of the process, the NiO-CoO/CC material will be separated. Figures 12B–I show the SEM image of nanowires of CoO on the CC with an average radius of 25–50 nm. Figure 12B (ii–iii) shows the morphology of NiO-CC at different magnifications, which confirms that there is a regular interconnect between the wires and is highly dense, which means the active material has a larger contact area with the electrolyte, boosting the electrochemistry of the contact area. Figure 12B (iv–vi) shows that CoO is grown homogeneously on the surface of NiO, which confirms that the 3D porous heterostructure offers active sites for redox reactions and helps the transmission of ions and electrons, which cause the enhancement of EC presentation.

An ASSC is fabricated by connecting the NiO-CoO/CC positive and negative electrodes of AC. Figure 12C shows that at a low voltage range, the CV graph appears to have a larger peak with a large CV area. On the other hand, the high voltage area of CV and redox peaks decreases slowly, possibly due to the incomplete reaction of active substances occurring at high voltage and large scan rates. Figure 12D shows a graph of GCD that explains that increasing current density causes an increase in charging and discharging time due to the ability of NiO to provide the good transportation of ions to promote the activity of CoO. Figure 12E shows the presentation of the fabricated device. It has been confirmed that with increasing cycles, the retention of the device decreases. After 5 K cycles, the device’s retention was 88%, and after 15 K cycles, the retention was 72%.

Figure 12F displays the Nyquist plot of the fabricated device with the circuit and the fitting curve before and after cycling. It has been seen that the resistance (R_2_ and R_3_) of the sample before cycling is smaller (0.19 and 0.01 Ω) than that after cycling (0.23 and 0.29 Ω). The sample has low resistance before cycling, which may be due to the contribution of active substances in the redox reaction and the increased interaction of electrolytes with the sustained substrate, which contains more active sites. The ED and PD of the NiO-CoO/CC are present in Figure 12G. The fabricated device shows an ED of 48 Wh/kg with a PD of 375 W/kg and also exhibits a high PD of 3 k W/kg of the device at an ED of 14 Wh/kg at low CD. On the other hand, Figure 12G shows that the three pictures that explain the ASSC can stand the large distortion and are sufficient for everyday applications. Figure 12H shows the ASSCs powering the programmable LED that provided the high current for 10 s after charging. The results explain that the fabricated device can be commercially applied for EESDs due to its high supercapacitive properties (Li et al., 2020). Ternary MO (Zn-Ni-Co) was prepared using the hydrothermal method and employed as an electrode material. In the fabricated device, the prepared material was used as the positive electrode and AC as the negative electrode, which showed that Zn–Ni–Co exhibits excellent specific capacitance (2,482 F/g), cyclic stability (94%), and high ED (35.6 Wh/kg). The notable EC performances prove that ternary MO (Zn–Ni–Co) electrode nanowires are highly desirable for advanced SC applications (Wu et al., 2015).

**FIGURE 13:**
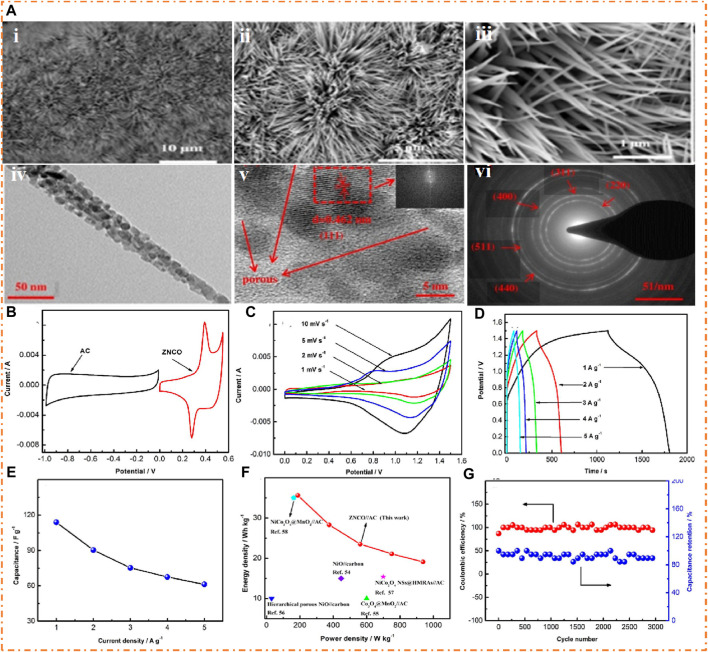
**(A)**-i–iii Needle-like SEM images of Zn-Ni-Co on Ni foam, **(A)**-iv TEM image of Zn-Ni-Co and **(A)**-v HRTEM image of a Zn-Ni-Co needle, and **(A)**-vi SAED pattern of the Zn-Ni-Co needle. CV curves of **(B)** AC and Zn-Ni-Co electrodes, **(C)** Zn-Ni-Co//AC ASSC, **(D)** charging and discharging curves, **(E)** specific capacitance, **(F)** Ragone plots of the Zn-Ni-Co//AC ASSC device, and **(G)** Coulombic efficiency and capacitance retention. Reprinted with permission from Wu et al. (2015).

Figure 13A(i–iii) shows the SEM images of ternary MO (Zn-Ni-Co) electrode material at low magnification, which showed a needle-like morphology with an average radius of 20–60 nm and 2–3 µm length. They employed different substrates like Ni foam, CC, Cu foam, and Si wafer, which showed the same morphology as the needle for all substrates. In all substrates, these needles are distributed uniformly in three dimensions on the substrate which provides a good pathway for ions and electrons and permits mass and charge exchange during the Faradaic redox reaction. Figure 13 (a–iv) shows the needle-like morphology of ternary MO (Zn-Ni-Co), also confirmed by the TEM, which showed a clear porous structure of the needle that contains nanocrystals with an average size of 2–10 nm. This exceptional morphology is demonstrated to be helpful for the penetration of electrolytes in the solution and the fast transfer of ions and electrons that causes improved electrochemical reactivity (Hu et al., 2012; Li et al., 2013). In Figure 13A (v), the interplanar distance (0.42 nm) and plane (111) of the Zn-Ni-Co electrode were measured by high-resolution TEM. In Figure 13A (vi), the well-shaped ring of Zn-Ni-Co was observed by SAED, which shows that the prepared electrode contains many crystals. The CV curve of the Zn–Ni–Co electrode and AC electrode in a rectangular form, exhibiting properties like EDLC, is shown in Figure 13B. The two peaks in the Zn–Ni–Co electrode explain the Faradaic behavior of the material offered. The specific capacitance of ASSC can be subsidized by the combination of Faradaic behavior and the ELDC of the material. There is almost no change in the shape of the CV curve with an increase in the scan rate, as shown in Figure 13C, which explains that the ASSC display has excellent capacitive behavior regarding increasing scan rate. The charging–discharging curves of the fabricated (Zn-Ni-Co//AC) ASSC device are given in Figure 13D, which are symmetric, representing the decent EC actions of the Zn-Ni-Co//AC ASSC device. The specific capacitance reached at 114 F/g at 1A/g of the Zn-Ni-Co//AC hybrid device is shown in Figure 13E.

**FIGURE 14:**
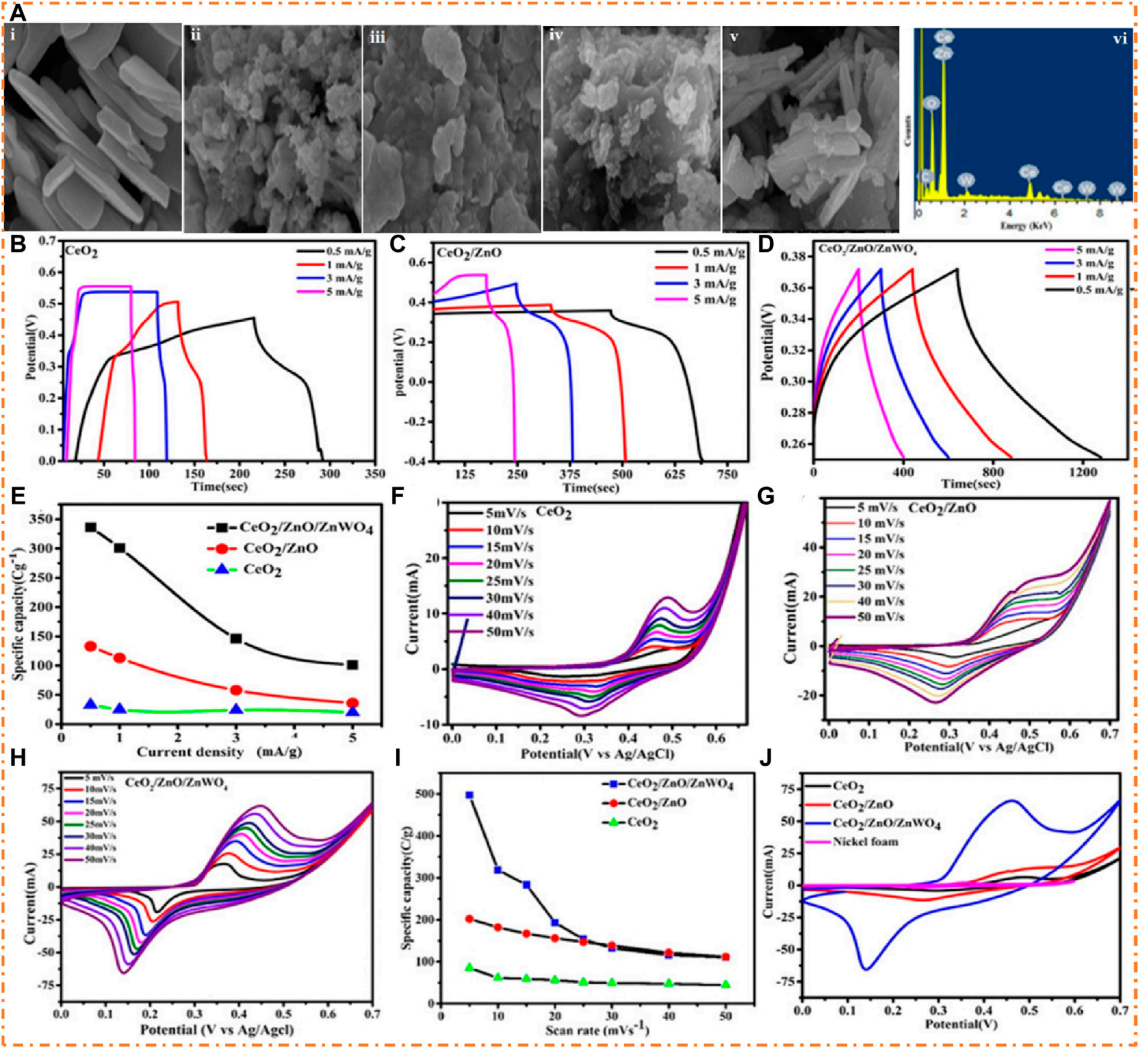
SEM images of **(A)**-i single CeO_2_, **(A)**-ii single ZnO, **(A)**-iii single ZnWO_4_, **(A)**-iv binary oxide (CeO_2_/ZnO), **(A)**-v ternary oxide (CeO_2_/ZnO/ZnWO_4_), and **(A)**-vi EDX CeO_2_/ZnO/ZnWO_4_. GCD curves of **(B)** CeO_2_, **(C)** CeO_2_/ZnO, and **(D)** CeO_2_/ZnO/ZnWO_4_. **(E)** Specific capacity vs. current density and CV curves at a scan rate of 30 mV/s, **(F)** single CeO_2_, **(G)** binary composites of CeO_2_ and ZnO, **(H)** composites of CeO_2_, ZnO, and ZnWO_4_, **(I)** specific capacitance, vs scan rates, and **(J)** CV plots of three materials with Ni foam. Reprinted with permission from Khawar et al. (2022).

The Ragone plot showed that the ASSC has a PD of 938 W/kg at an ED of 19 Wh/kg at low CD and an ED of 36 Wh/kg at a PD of 188 W/kg at high CD, and the comparison with the literature is shown in Figure 13F. Single metal oxides with AC (NiO//carbon) showed an ED of 15 Wh/kg at a PD of 447 W/kg). Figure 13G shows how the cycling presentation of the Zn-Ni-Co//AC ASC device is demonstrated after measuring it for 3,000 cycles. The results show that capacitance retention will remain at 94% and Coulombic efficiency will remain at 100% during the entire measurement period. Khawar et al. (2022) prepared the MO (CeO_2_ loaded with ZnO and ZnWO_4_) and tertiary composites of oxide (CeO_2_/ZnO/ZnWO_4_) using the hydrothermal method. They investigated the morphological and electrochemical properties of the material. The SEM images show ZnO and ZnWO_4_ cover sheets of CeO_2_, and the measured capacity of the electrode was 497 C/g. The fabricated device exhibits a high PD of 2,000 W/kg at 56.92 Wh/kg ED and also has good stability (88%). The SEM images of CeO_2_ (single oxide), CeO_2_/ZnO (binary oxide), and CeO_2_/ZnO/ZnWO_4_ (ternary oxide) are shown in Figure 14. Figure 14A (i) shows that the nanosheets of CeO_2_ are oriented randomly in various directions. Figure 14A (ii, iii) shows the agglomerated particles of ZnO and ZnWO_4,_ respectively. In ZnO/CeO_2_, the particles of ZnO are loaded on the surface of CeO_2_ with a flake-like structure, as shown in Figure 14A (iv). On the other hand, the surface of a sheet of CeO_2_ is decorated by ZnO and ZnWO_4_, which proves the formation of a large surface area of the material for oxidation and reduction reactions, as shown in Figure 14A (v). Using EDX, the chemical composition of CeO_2_/ZnO/ZnWO_4_ is determined and is shown in Figure 14A (vi).

Figures 14B–D show the triangular shape of CeO_2_/ZnO/ZnWO_4_, which confirms the battery-like behavior of the material. Figures 14B–D show the GCD curve of CeO_2_, CeO_2_/ZnO, and CeO_2_/ZnO/ZnWO_4_ at various current densities (0.5–5 mA/g). Figure 14C shows the rectangular shape of CeO_2_, and Figure 14D shows the rectangular shape of binary composites (CeO_2_ and ZnO). There have been observations concerning the redox potential change that occurs when the CD increases, which may also be attributed to the material’s polarization and ohmic contributions. The formation of rectangular CV curve shapes is due to polarization and ohmic contributions. Figure 14E shows the graph of specific capacity inversely related to the current density. The decreasing trends in specific capacitance are due to the fast reaction at the surface of the electrode materials, which decreases the ion diffusion at high current density.

The CV plots of CeO_2_, CeO_2_/ZnO, and CeO_2_/ZnO/ZnWO_4_ of electrode materials with various SR (5–50 mV/s) show the pseudocapacitive behavior, as shown in Figures 14F–H. Figure 14F confirms that double redox peaks are due to the presence of two oxidation states of cerium. Figure 14G shows that the composite areas are increased compared to single oxide, which is the incorporation of Zn and Cr ions. Figure 14H shows the maximum redox current obtained at 50 mV/s with the unchanged shape of the CV curve. With increasing SR, the value of Cs of three electrode materials decreases, as shown in Figure 14I. The CV plots of three electrode materials deposited on the Ni foam are also demonstrated in Figure 14J. It is clear that Ni foam does not influence the charge storage process at the given scan rate and acts only as a current collector, while the deposited material only responds to CV signals.

**FIGURE 15:**
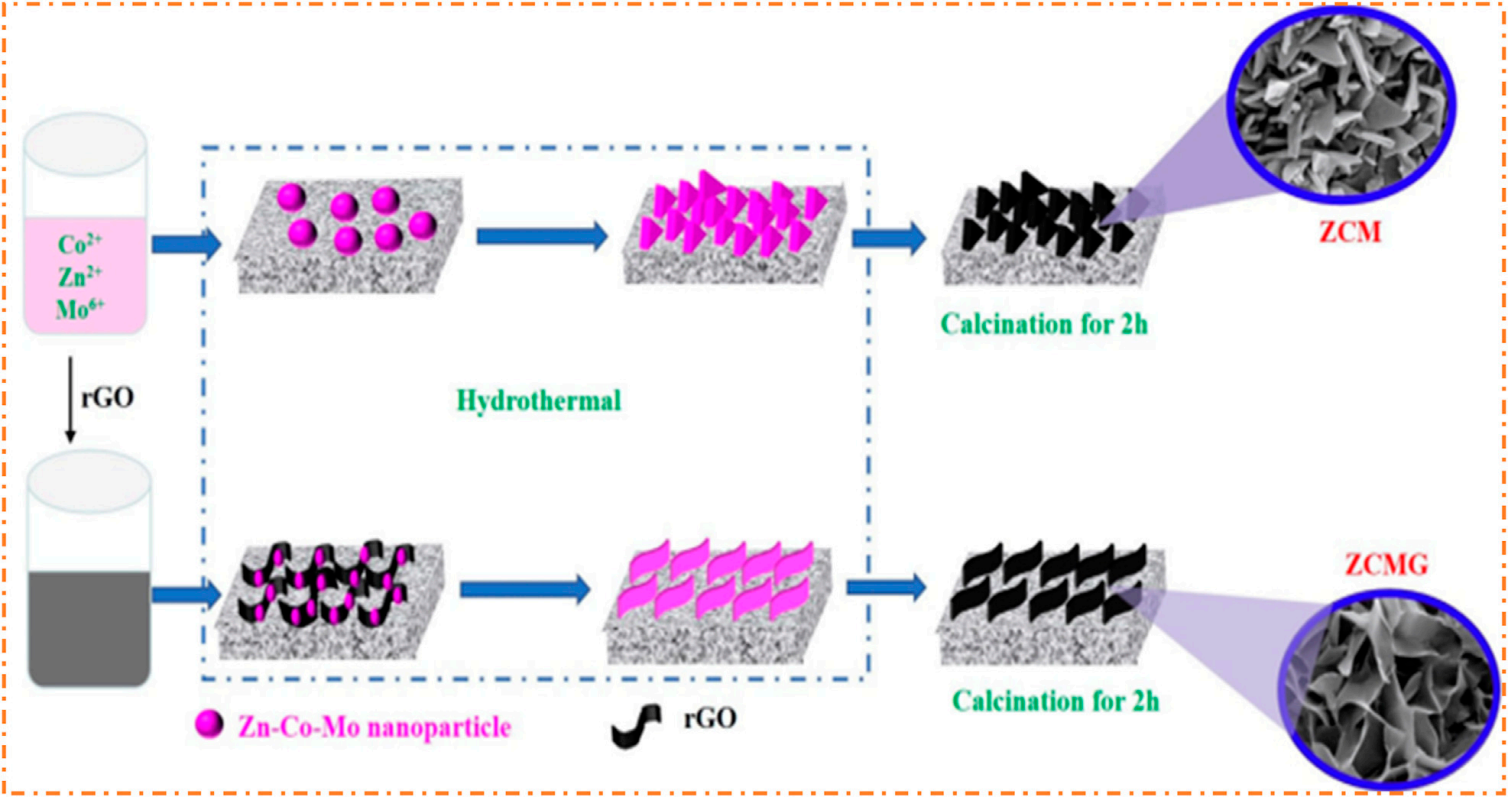
Synthesis route of Zn/Co/Mo and Zn/Co/Mo/rGO nanosheets on NF. Reprinted with permission from Liu et al. (2023).

Liu et al. (2023) observed that the composites of ternary MO (Zn-Co-Mo) electrodes prepared by the hydrothermal method have a low specific capacitance of 492 F/g compared to the composite of the electrode of Zn-Co-Mo with rGO, which has a high specific capacitance of 1,189 F/g. The gathered electrode of Zn-Co-Mo-rGO and AC, ASSC, carries 68 C/g specific capacity. This device has achieved a high-capacity retention rate of 95% after 1,000 cycles of operation. Additionally, the ASSC has an ED of 5.23 Wh/kg at a PD of 7,500 W/kg. The simple synthesis route for the preparation of ternary oxide Zn-Co-Mo and the composite of ternary composites Zn-Co-Mo with rGO is shown in Figure 15, which shows a different morphology.

**FIGURE 16:**
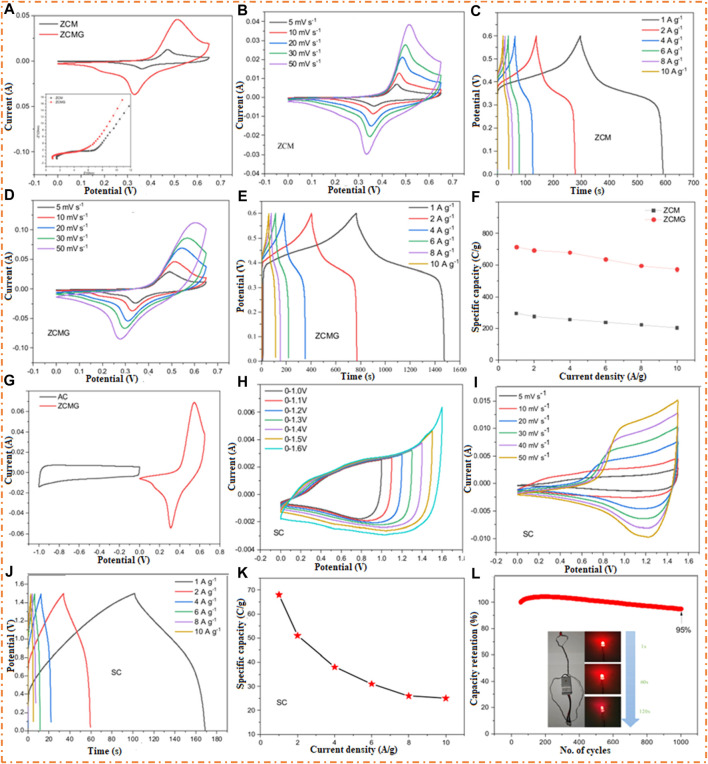
**(A)** CV plots of Zn/Co/Mo and Zn/Co/Mo/rGO (inset of the N-plots of Zn/Co/Mo and Zn/Co/Mo rGO. **(B)** CV plots of Zn/Co/Mo. **(C)** GCD plots of Zn/Co/Mo. **(D)** CV plots of Zn/Co/Mo/rGO. **(E)** GCD plots of Zn/Co/Mo/rGO. **(F)** Specific capacity plots of Zn/Co/Mo and Zn/Co/Mo/rGO and **(G)** CV plots of Zn/Co/Mo/rGO and AC electrodes. **(H)** Plots of CV of ASSCs at different voltages. **(I)** Plots of CV of ASSCs at several scan rates. **(J)** GCD plots of ASSCs. **(K)** Specific capacity plots of ASSCs. **(L)** Cycling stability of ASSCs (inset shows the physical presentation). Reprinted with permission from Liu et al. (2023).

The electrochemical properties of Zn/Co/Mo and Zn/Co/Mo/rGO were studied by the 3E system in 2 M aqueous KOH. Figure 16A represents the CV profile of Zn-Co-Mo and Zn-Co-Mo-rGO with varying voltage and scan rates. It shows that at a high scan rate, the area of the CV profile of Zn-Co-Mo-rGO is higher than that of Zn-Co-Mo, which concludes the higher specific capacitance. The CV curve of Zn/Co/Mo at various scan rates is given in Figure 16B, and the CV curve of Zn/Co/Mo/rGO at different scan rates is presented in Figure 16D. It is clear that when the scan rate was increased from 5 to 50 mV/s, the redox couples experienced a shift in their reduction and oxidation peaks toward the low and high potential directions due to the polarization of the electrode (Cao et al., 2016). Moreover, if you consider that the shape of the CV curve does not change in response to an increase in the scan rate, this implies that Faradaic reactions can be reversible quickly with a good rate of capability.

At different current densities, the GCD plots of prepared Zn/Co/Mo and Zn/Co/Mo/rGO are shown in Figures 16C,E, respectively. The GCD curves present a charge/discharge plateau closely related to the redox peak on CV plots. In contrast, the symmetrical curves of GCD denote the reversible process, which can be described as a Faradaic redox reaction. On the other hand, the specific capacity of Zn/Co/Mo is lower than that of Zn/Co/Mo/rGO, as shown in Figure 16F, which may be due to the formation of a thin sheet of material that enhances the property of the material. For oxidation and reduction reactions, the construction of a sheet provides a better result, such as the greater surface area of the sheet providing more active sites and excellent charge storage capacity. When electrons are transported through the thin sheet during charge/discharge, the electron diffusion between the electrolyte and active material becomes faster. The Nyquist plots of both materials are shown in the inset of Figure 16A. The Nyquist plot has two parts: nearly straight lines denoting the low value of frequency and a semi-circle representing the high-frequency value. Additionally, the slope of Zn/Co/Mo/rGO is higher than that of Zn/Co/Mo, which causes lower ionic diffusion resistance. The small resistance value of a thin sheet of Zn/Co/Mo/rGO provides high diffusion of ions and fast electron transfer. The operability of Zn/Co/Mo/rGO as an electrode for ASC was obtained using Zn/Co/Mo/rGO as the positive electrode and negative electrode of AC. The CV plots of Zn/Co/Mo/rGO and AC are given in Figure 16G. The CV plots of the ASSC are displayed in Figure 16H, which show the irregular behavior of the curve with increasing voltage, possibly due to electrolyte decomposition.

Figure 16H displays the CV plots at several scan rates. The shape of CV plots is unchanged as the scan rate changes, which proves the good stability rate. Figure 16I shows the GCD plots at several CDs and the nearly symmetric shape, which confirms the good Coulombic efficiency of the ASSC. The specific capacity is inversely proportional to CD, as shown in Figure 16J. The decrease in particular capacity at high current density is due to the increased diffusion resistance of ions and the low penetration power of electrolytes (Naeem et al., 2020). The main factor for checking the fabricated device is cyclic stability, which is 95% under 1,000 cycles, as shown in Figure 16K. The R-plot (Ragone plot) of ASSC devices explains the PD of 750 W/kg at an ED of 14 Wh/kg and also finds an excellent PD of 7,500 W/kg at an ED of 5.23 Wh/kg.

**FIGURE 17:**
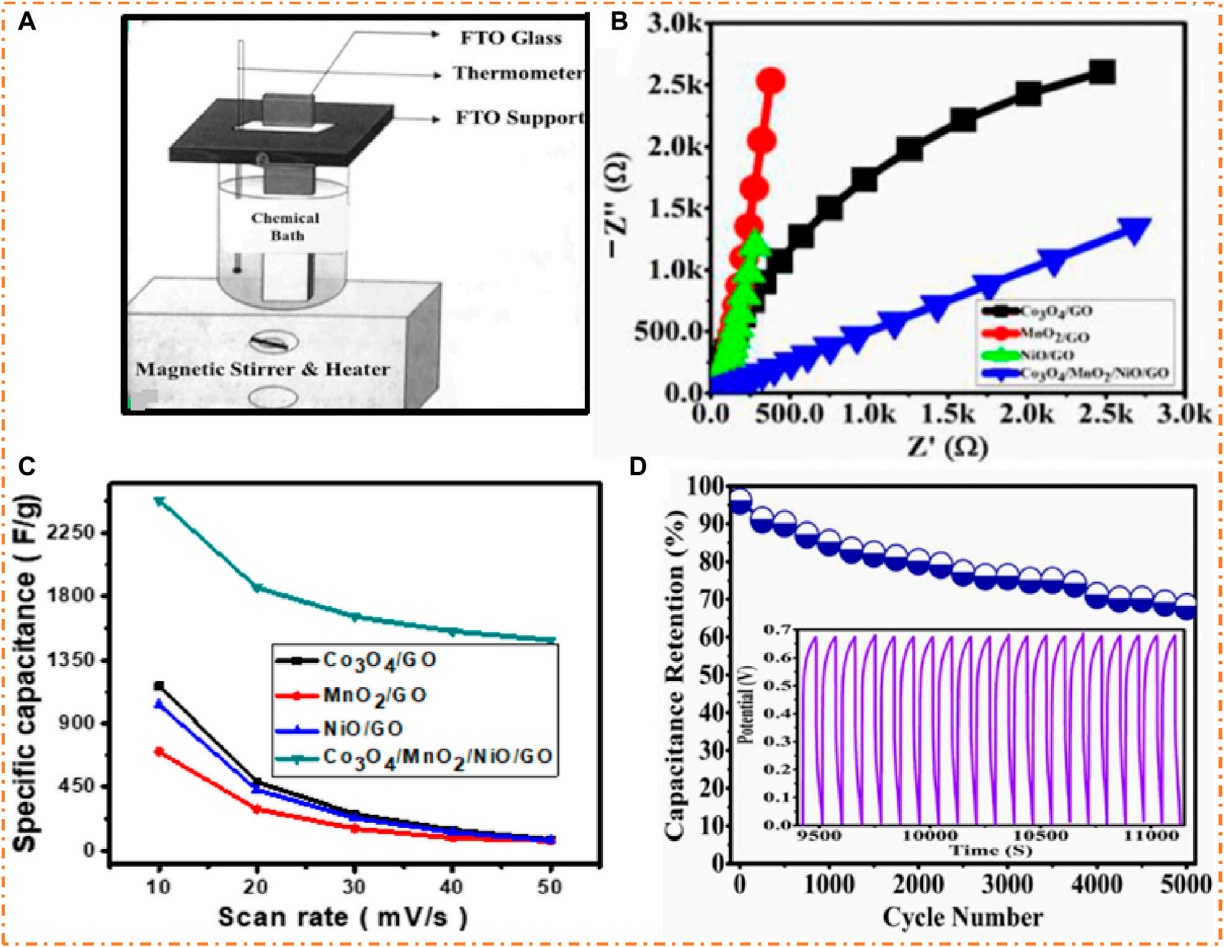
**(A)** Setup of chemical bath deposition for electrode preparation, **(B)** EIS of the cobalt oxide with GO, manganese oxide with GO, nickel oxide with GO, and Co-Mn-Ni-O with GO electrodes, **(C)** SC vs SR, and **(D)** stability test of the Co-Mn-Ni-O/GO electrode. Reprinted with permission from Alshoaibi et al. (2022).

Alshoaibi et al. (2022) used the chemical bath deposition process to synthesize the nanocomposites of TMO with GO for SC applications, as shown in Figure 17A. Incorporating GO with single MO electrodes enhances performance and stability. The CV results confirm that the material has a good specific capacitance of 1,166 F/g for Co-GO, 699 F/g for Mn-GO, 1,032 F/g for Ni-GO, and 2,482 F/g for Co-Mn-Ni-O with GO at 10 mV/s. The EIS of the cobalt oxide with GO, manganese oxide with GO, nickel oxide with GO, and composites of Co-Mn-Ni-O with GO electrodes is shown in Figure 17B, which confirms that composite oxides with GO electrodes (Co-Mn-Ni-O with GO electrodes) have excellent results as compared to single MOs with GO electrodes, indicating a synergistic relation between them. The retention changed very slowly, representing the cyclic and reversible systems, and SC’s highest value was Co-Mn-Ni-O composites with GO electrodes, as shown in Figure 17C. Compared with single MOs and bi-metallic oxides (BMOs), in ternary MO with Go and rGO, more elements contributed to the oxidation and reduction reactions, showing high EC activity. It has been noted that the addition of GO and rGO causes an enhancement in the electrical conductivity and cycle presentation of fabricated materials. Co-Mn-Ni-O with GO electrodes with better features was at CD of 1 A/g, showing 96.5% cycling stability after 5 K cycles, as shown in Figure 17D. The GCD plots of the last 20 cycles showed minimal fluctuations, indicating outstanding cyclic stability. A drop of about 30% in the Co-Mn-Ni-O with GO electrode’s capacitance could be due to the decrease in the electrode’s active mass during prolonged redox activity. In this study, the finding values of E_d_/P_d_ were 243 Whkg^−1^/16.2 Whk/g for cobalt oxide with GO, 304 Whkg^−1^/21 Wkg^−1^ for manganese oxide with GO, 153 Whkg^−1^/10.4 Wkg^−1^ for nickel oxide with GO, and 335 Whkg^−1^/32.2 Wkg^−1^ Co-Mn-Ni-O with the GO electrode.

In summary, although carbon materials have an elevated power density, a low value of energy density limits their overall efficiency. Compared to carbon materials, TMOs offer a larger specific capacitance, an elevated energy density, and superior chemical stability over conductive polymers (Khawar et al., 2022; Manasa et al., 2022). Materials such as carbon materials, conducting polymers, and MOFs can be mixed with other materials to create hybrid electrode architectures that improve the electrochemical performance of metal oxides. These hybrid materials typically show synergistic effects, combining the benefits of both to increase electrochemical activity through increased electrical conductivity, cycle stability, and capacitance. On the other hand, the better results of MOs with the carbon-based material are due to the greater surface area of the electrode material, which provides more active sites and excellent charge storage capacity.

From this study, it has been found that monometallic oxide (Sethi et al., 2021) has lower electrochemical performance than a composite of metal oxide with carbon base materials (Li et al., 2021). Bimetallic oxide has lower electrochemical performance than composites of binary metal oxide with carbon-based electrode materials (Chen et al., 2020b; Li et al., 2020). Similarly, trimetallic oxide (Khawar et al., 2022) has lower electrochemical performance than composites of tri-metal oxide with carbon base electrode materials (Alshoaibi et al., 2022). In both bi- and tri-metal oxides, the synergistic effects of elements are present, but when comparing the tri-metal oxides to bi-metal oxides, bi-MOs have fewer active sites, lower synergistic effects, and lower electrical conductivity than the tri-MO. This study shows that the electrical conductivity of the oxide materials (mono/bi/tri-metal oxide) is higher than that of the carbon material but has a lower surface area. The composite of metal oxides with carbon-based materials yields excellent electrochemical performance. It has been suggested that, as compared to simple metal oxides (mono/bi/tri-metal oxides), composites of MOs with carbon have more distributed redox-active sites and a larger surface area, resulting in reduced electrical resistivity, increased redox current, and improved electrochemistry.

## 4 Composites of MOs with conductive polymer-based electrode materials

A new class of materials employed for the fabrication of electrode materials is the composites of MOs with CPs (Patil et al., 2022). We know that an SC offers high specific power and excellent cycling stability, which are the main parameters of a supercapacitor. It has been considered that the electrode fabrication from MOs and CPs enhanced the electrochemical presentation of SCs. Researchers are investigating various MOs with CPs today, offering better electrochemical performance (Abdah et al., 2020; Ahmed et al., 2023). While MOs and CPs have advantages and limitations, their composites may provide some additional information about the properties of electrode materials. Conducting polymers like PANIs have many properties, including high conductivity, ease of synthesis, and low cost. Still, CPs have lower mechanical stability and periods of charge–discharge, which make them unsuitable for SCs.

Compared with PPy, PEDOT has superior electrolytic contact but a minor mass and a non-porous structure. PPy’s non-porous structure may affect its cycling stability, and PEDOT’s porous structure may provide electrolytic contact but can impair cycling stability. However, MOs have good specific capacitances, are inexpensive, have a limited surface area, and have low electrical conductivity, making them difficult to use as electrode materials for SCs. The performance of SCs can be improved by using CPs and MOs as nanocomposites, as they can enhance surface area, ionic and electrical conductivity, specific capacitance, stability, and ED and PD (Wang et al., 2014; Liew et al., 2017; Low et al., 2019; Roy et al., 2020; Raza et al., 2022).

MnO_2_ is a promising electrode material due to its high abundance, nontoxicity, and ease of fabrication. MnO_2_ has outstanding EC properties, including a high conductivity and excellent performance in neutral electrolytes. Despite this, its actual specific capacitance is much inferior to its theoretical specific capacitance due to its weak conductivity, low structural stability, and low electron transport ability (Liu et al., 2016; Pan et al., 2016; Zhao and Wang, 2016; Yin et al., 2019; Li et al., 2022). Researchers have developed various nanonetwork MnO_2_/PANI (MP) composites, with the highest SC reaching 497 F/g and good cycle stability (Zhao and Wang, 2016). Liu et al. fabricated the ternary nanofibers with a good specific capacitance of 349 F/g and maintained 88.2% retention after 2 K cycles (Liu et al., 2016). Pan et al. (2016) prepared the PANI-MnO_2_/graphene composites with an SC of 695 F/g after 1 K cycles. Li et al. (2022) fabricated CC/MnO_2_/PANI nanofibers with an SC of 729 F/g and 87% retention after 2 K cycles. A composite of PEDOT/MnO_2_ obtained an SC of 366 F/g and had good retention after 2 K cycles (Yin et al., 2019).

**FIGURE 18:**
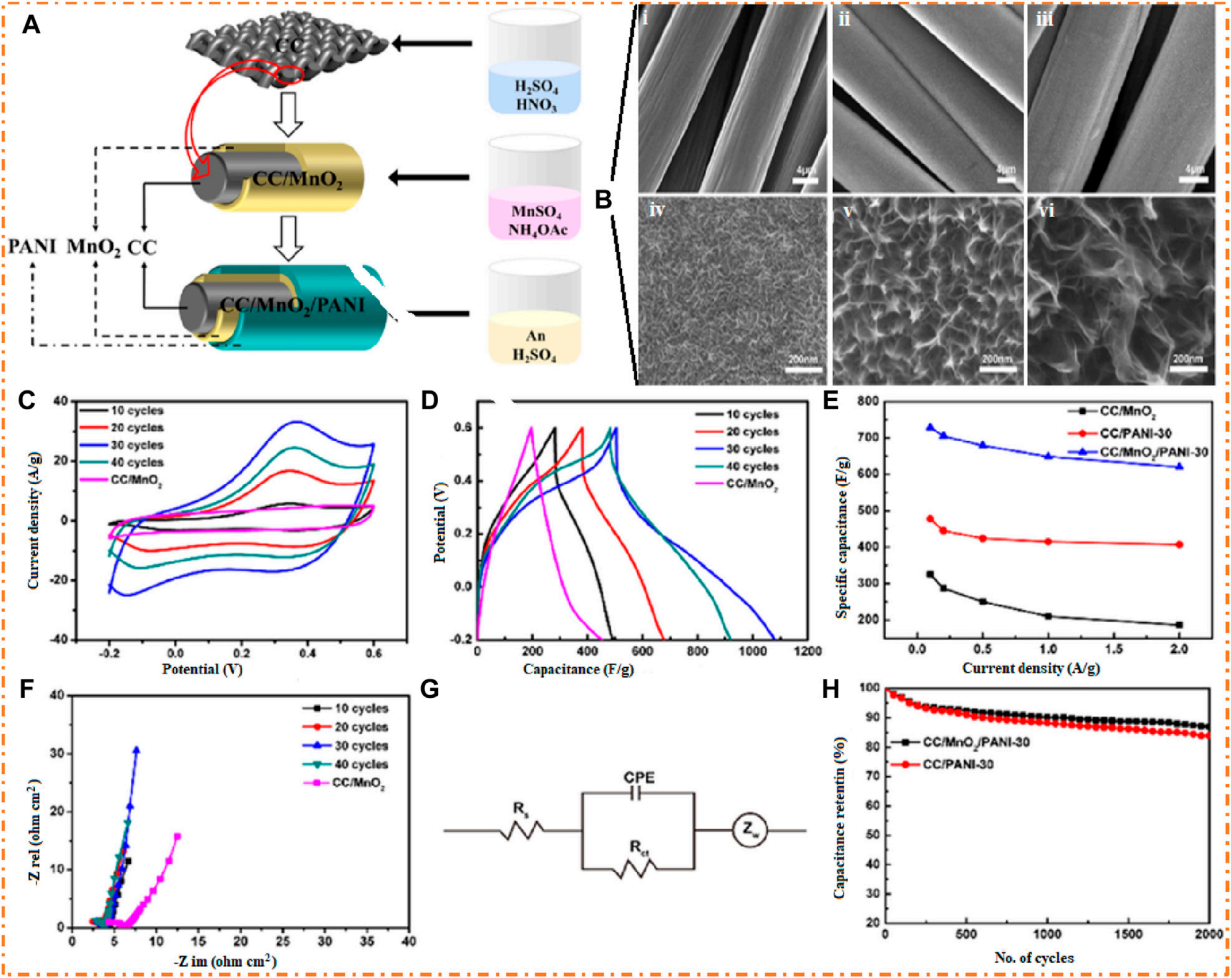
**(A)** Schematic diagram of the CC/MnO_2_/PANI composite and SEM images at deposition time: **(B)**-i and iv 10 min, **(B)**-ii and iv 20 min, and **(B)**-iii and v 30 min. **(C)** CV plots, **(D)** GCD plots, **(E)** specific capacitance, **(F)** Nyquist plots, **(G)** equivalent circuit, and **(H)** cyclic stability. Reprinted with permission from Li et al. (2022).

Li et al. (2022) prepared the ternary composites (CC/MnO_2_/PANI) using the electrodeposition process to increase the stability and capacitance, as shown in Figure 18A. They explain the EC performance of the electrode material as a function of the deposition time (0–30 min) of the distribution of particles on the surface. Figure 18B (i–vi) shows the shape of the fabricated material at different magnifications (4 micro b–d with time 10, 20, and 30 min and 200 nano e–g with time 10, 20, and 30 min), which confirms that with increasing time (from 10, 20, and 30 min) of deposition, the rods are arranged uniformly, and at time 30 min, the rods are uniformly distributed. The sample CC/MnO_2_/PANI-30 is rich in nanofibers and has uniform layered structures with a maximum SC 729 F/g, as shown in Figure 18E. The CV and GCD plots in Figures 18C,D confirm that sample CC/MnO_2_/PANI-30 shows a good area of the CV profile and good discharging time.

Figure 18F shows the Nyquist plots that confirm that the CC/MnO_2_/PANI-30 sample has the lowest resistance and high specific capacitance. The equivalent circuit diagram is shown in Figure 18G. The maximum cyclic stability of the CC/MnO_2_/PANI-30 sample is 87% after 2 K cycles, as shown in Figure 18H. NiO is a superior SC electrode due to its higher theoretical capacitance and high surface area. Its low fabrication price and lack of environmental impact make it a feasible substitute for other materials. Nevertheless, the lower electrical conductivity of this material and the smaller surface area result in low reversibility and limited capacitance when it comes to charging and discharging this material. To address this, researchers have developed porous/hollow designs to increase the active surface area of materials (Singu et al., 2016; Zhang J. et al., 2017). A nanocomposite of PANI-NiO has good specific capacitances of 514 F/g (Singu et al., 2016). It has been found that the nanofiber of AC-PANI/NiO composites for SCs displays high specific capacitance (1,157 F/g) and remarkable retention of 94% after 5 K cycles (Zhang J. et al., 2017). Han et al. (2019) found that NiO/PPy/AC, an asymmetric SC, has high Cs of 938 F/g and ED and PD of 333 Wh/kg and 2,400 W/kg (Han et al., 2019).

**FIGURE 19:**
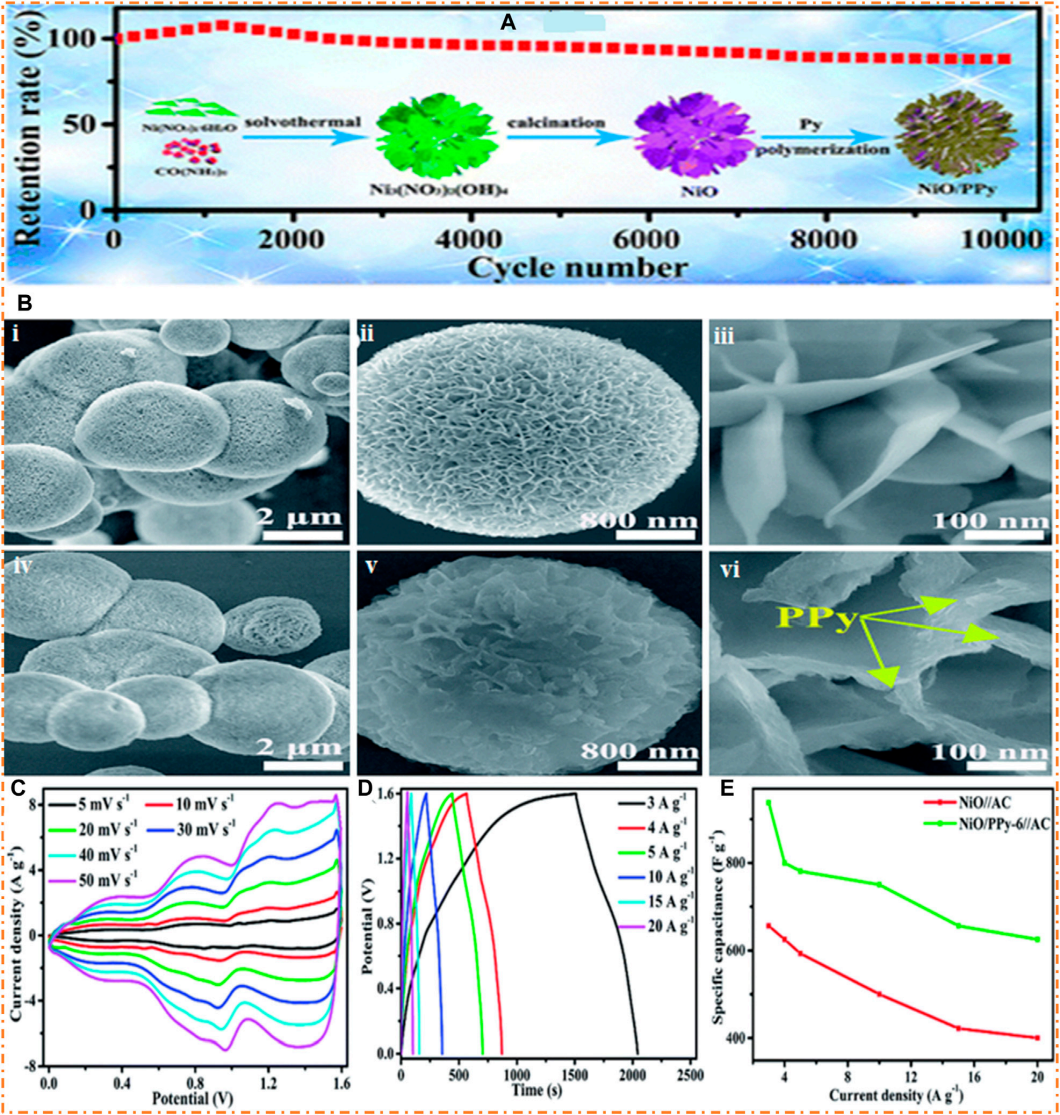
**(A)** Synthesis route of NiO/PPy and FESEM images at low and high magnification **(B)**-i–iii NiO, **(B)**-iv–v NiO/PPy, **(C)** CV plots, **(D)** GCD plots, and **(E)** specific capacitance. Reprinted with permission from Han et al. (2019).

Han et al. (2019) synthesized the NiO/PPy microspheres by polymerizing pyrrole on the nanosheets of NiO, which are surrounded by poly-pyrrole with a fish-scale-like shape, as shown in Figure 19A. In the first step, two materials, Ni(NO_3_)_2_·6H_2_O and CO(NH_2_)_2_, are subjected to alcohol-lysis slowly, resulting in the production of Ni_3_(NO_3_)_2_(OH)_4_ microspheres. In the second step, the microspheres of Ni_3_(NO_3_)_2_(OH)_4_ are calcinated to obtain NiO. The final step involves creating wrinkly PPy films on the surfaces of the NiO nanosheets by polymerizing Py, resulting in fish scale-like morphologies on the surface. The microspheres of NiO/PPy were employed as the electrode material in an SC. The high SC of 3,649 F/g and good rate capability were observed at high current density. NiO/PPy-6 prepared an ASC as the positive electrode and negative electrodes of AC, and NiO/PPy-6//AC can obtain a good SC and a high ED of 333 W h/kg at 2,400 W/kg PD. The prepared material has a high retention rate. The FESEM characterization of NiO and NiO/PPy microspheres is revealed at low and high magnification in Figure 19.

Figure 19B(i–iii), the NiO images clearly show the nanosheet-like morphology. The nanosheet of NiO exhibits space for the polymerization of Py and causes a reduction in length for the diffusion of ions in the electrolyte solution. Figure 19B (iv–vi) shows the images of composites (NiO/PPy) compressed structure and nanosheets. The CV plots of composites (NiO-PPy-6//AC) and alone (NiO//AC) at several scan rates are shown in Figure 19C. The CV curve remains stable as the scan rate increases, suggesting good cycling performance and a satisfactory scan rate. The GCD plots of composites (NiO/PPy-6//AC) and alone (NiO//AC) at the several current densities are shown in Figure 19D. As shown in Figure 19E, the composites NiO/PPy-6//AC have a high Cs of 938 F/g, greater than those of single NiO//AC (656.2 F/g).

**FIGURE 20:**
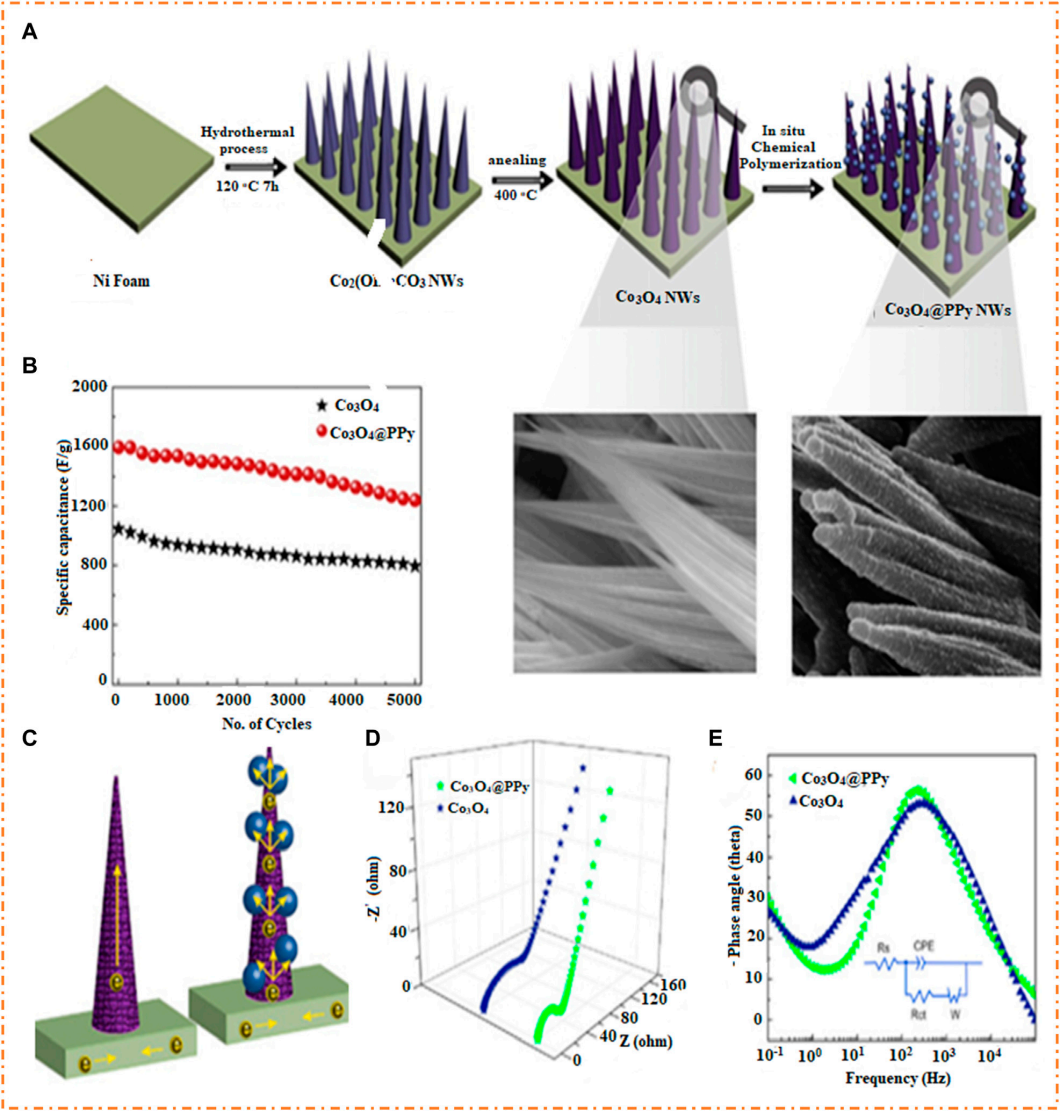
**(A)** Synthesis route of core-shell nanowires Co_3_O_4_/PPy on the surface of Ni foam, **(B)** cycling stability of Co_3_O_4_/PPy and Co_3_O_4_, **(C)** pictorial illustration for the transfer of electron using both Co_3_O_4_ and PPy provide channels, and plot of Co_3_O_4_/PPy and Co_3_O_4_ electrodes. **(D)** Nyquist plot and **(E)** Bode plot with an inset picture of equivalent circuit. Reprinted with permission from Guo et al. (2017).

Co_3_O_4_ is a proficient SC material due to its strong redox characteristics, high capacitance, simple synthesis, and suitability as an electrode material substitute. Significant efforts have been focused on developing nanostructured Co_3_O_4_ to improve electrochemical performance due to low conductivity, slow kinetics, and particle aggregation (Hai et al., 2016; Guo et al., 2017; Ren et al., 2018). Recent studies have shown that hierarchically hollow Co_3_O_4_/polyaniline nanocages have a large Cs (1,301 F/g), ED (41.5 Wh/kg), PD (1,600 W/kg), and good stability of 90% after 2 K cycles (Ren et al., 2018). On the other hand, core-shell PANI- Co_3_O_4_ nanocomposites have a good Cs of 1,184 F/g and superior cycling stability of 85% after a 1 k cycle (Hai et al., 2016). Hierarchical Co_3_O_4_@PPy core-shell composite nanowires have also shown high specific capacitance (2,122 F/g) and cycling stability of 78% after 5 k cycles (Guo et al., 2017). Sulaiman et al. (2017) prepared a nanocomposite PEDOT/GO/Co_3_O_4_ with a Cs of 536 F/g and capacitance retention of 92.7% after 2 k cycles. With the help of a two-step route, Guo et al. (2017) fabricated the core-shell Co_3_O_4_/PPy, as shown in Figure 20. In the first step, the nanowires of Co_3_O_4_ are manufactured on the surface of Ni foam by hydrothermal and post-annealing methods. In the second step, the PPy film was coated on the nanowires of Co_3_O_4_ by polymerization, and composites of Co_3_O_4_/PPy were formed, as shown in Figure 20A.

The composites of Co_3_O_4_/PPy nanowires have a high SC of 2,122 F/g, which is greater than a single Co_3_O_4_ nanowire, and also have good cycling stability with 78% after 5 K cycles, as shown in Figure 20B. It has been noted that by using PPy, the charge transfer resistance could be significantly reduced and the active sites could be increased. Figure 20C shows that the hybrid core-shell structure is believed to enhance electrode performance by allowing easy diffusion of electrolyte ions, providing fast electron transfer pathways, and allowing open space between the Co_3_O_4_ nanowires. The composite Co_3_O_4_/PPy exhibits lower equivalent series resistance than pure Co_3_O_4_ due to its unique electrode structure, which facilitates rapid charge transfer and exhibits a sharper slope in the low-frequency region, as shown in Figure 20D. Figure 20E shows the Bode plots of the Co_3_O_4_/PPy and Co_3_O_4_ electrodes, which confirm the pseudocapacitive nature of the Co_3_O_4_/PPy electrode, ascribed to its rational hierarchical heterostructure design. The PPy film increases surface area, improves electronic conductivity, and allows for fast electron transportation, allowing the full contribution of the Co_3_O_4_ NWs in the Faraday redox reaction.

Vanadium-based oxides are a promising supercapacitive electrode due to their various oxidation states and large potential windows. They are naturally occurring, non-expensive, and have high specific capacity but have low conductivity and structure stability (An et al., 2019). The composites of V_2_O_5_-PANI NWs have a maximum Cs of 443 F/g, an ED of 69.2 Wh/kg, a PD of 7.2 kW/kg, and good stability with 92% capacitance retention after 5 K cycles (Asen et al., 2017). Reported research on the nanocomposite of V_2_O_5_/PPy/GO showed that it generated a Cs of 750 F/g, an ED of 28 Wh/kg at a PD of 3.6 kW/kg, a PD of 13.7 kW/kg at an ED of 23 Wh/kg, and cyclic stability of 83% after 3 K cycles (Prasanna et al., 2017). Iron oxide is attractive since it has a wide operating potential, a high redox activity, low cost, diverse crystallographic forms, and is abundantly available. Prasanna et al. prepared nanocomposites (PANI/Fe_2_O_3_), which showed high Cs (974 F/g), ED (118 Wh/kg), PD (9,800 W/kg), and good cycling stability (94%) after 2 K cycles (Sahoo and Shim, 2017).

In summary, this study demonstrates that CP and MO composites are widely investigated as electrode materials in SCs. CPs have poor cycling stability and rigidity but are simple to prepare, as well as reversed surface-redox processes and pseudocapacitive characteristics, whereas MOs are readily available, have a variable oxidation state, excellent stability, adaptable assets, and an elevated specific capacitance, but are a little harder to synthesize. As a consequence, composites containing combined CPs and MOs provide superior electrochemical performance for supercapacitors. On the other hand, it has been noted that carbon-based materials are employed in SCs, which provide high specific capacitance, high conductivity, porous structure, and improved stability as compared to pristine nature. These materials’ structural instability and low electrical conductivity limit their application fields. The continued study in this area will reveal additional potential applications for composites of CPs with MOs and CPs with carbon-based electrode materials.

Though the reported electrode materials in the recent review are remarkable developments in the energy storage field, the low value of electrical conductivity and low surface area of these materials usually lead to a low value of capacitance, a low performance rate, and restricted cycle stability throughout the redox process. These materials are not suitable for widespread usage in the manufacturing of industrial supercapacitors because of their inherent limitations. From this point of view, the development of a novel composite electrode material with favorable electrochemical characteristics and a synergistic effect between different electrode materials is therefore of significant interest to many scientific researchers. Therefore, scientists have applied several methods to improve the morphology and structure of currently available materials in order to satisfy the requirement for high-performance supercapacitors. So it has been noted that there are some ideas that can enhance the electrochemical properties of materials, like doping of materials, composition of different materials in composites, core-shell-like structures, and hierarchy.

**FIGURE 21:**
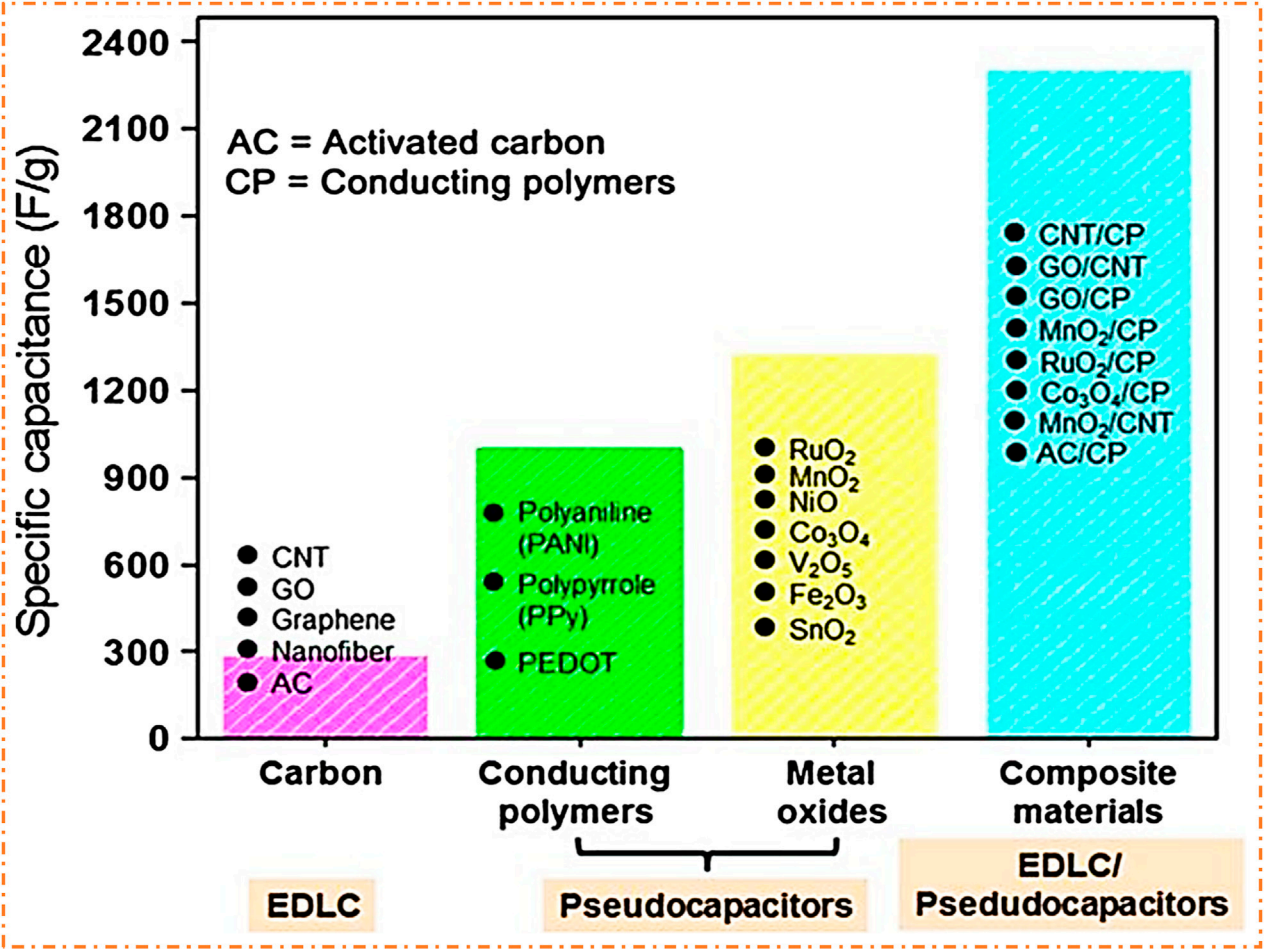
Specific capacitance of single-carbon-based materials, CPs, MOs, and their composites (MOs/CPs). Reproduced with permission from Shown et al. (2015).

The composites of oxides have good electrochemical performance but are expensive and difficult to manage on a large scale. It is important to note that the performance and characteristics of oxide and their composite-based SCs can vary significantly depending on the specific electrode materials, electrode designs, and overall device configurations. Ongoing research and development aim to optimize these SCs further, improve their energy storage capabilities, and expand their practical applications. However, their limits are still present commercially. The comparison of single MOs, CPs, and their composites is shown in Figure 21 (Shown et al., 2015). The measured values of specific capacitance, PD, and ED and methods of preparation of MOs and their composites based on electrode materials for SCs are given in Table 1.

**TABLE 1:** Electrode materials, method of preparation, morphology, electrolytes, scan rate, current density, specific capacitance, power density, energy density, and fabricated device retention of oxide-based electrode materials.

MOs	Method	Morphology	Electrolyte	SC (F/g)	CD (A/g)	ED (Wh/kg)	PD (W/kg)	Device	Retention (%)/no. of cycles	Reference
Co_3_O_4_	Solvothermal	Nanosphere	1 M Na_2_CO_3_	214.7	1	68.7	500	SCs	—	Jennifer et al. (2022)
NiO	Hydrothermal	Flake	2 M KOH	568.7	0.5	52.4	800	ASSC	90/5,000	Vinodh et al. (2022)
NiO	Solvothermal	Nanoflake	2 M KOH	305.0	10	1.20	8,000	SSC	95/5,000	Sethi et al. (2021)
rGO@Co_3_O_4_/CoO	Microwave	NPs on nanosheet	0.1 M KOH	276.1	5	—	—	SCs	82.4/10,000	Kumar et al. (2021b)
NiO/rGO	Hydrothermal	Nanosheet	2 M KOH	435.25	1	76.96	400	HSC	68/25,000	Li et al. (2021)
NiO/ZnO	Sol–gel	Porous NP	1 M Na_2_SO_4_	469	1 m	91.14	1,458.33	SCs	—	Anandhi et al. (2019)
ZnCo_2_O_4_	Solvothermal	Porous NP	2 M KOH	804	1	860.1	34.4	ASSC	79.2/3,000	Chen et al. (2020b)
NiO-CoO/CC	Hydrothermal	Needle-nanosheet	1 M KOH	1,024.05	1	40.3	750	ASSC	71.7/15,000	Li et al. (2020)
ZnNiCoO	Hydrothermal	Nanowire	6 M KOH	2,482.8	1	35.6	938	ASSC	94/3,000	Wu et al. (2015)
CeO_2_/ZnO/ZnWO_3_	Hydrothermal	Nanosheet	2 KOH	496.9	0.5 m	56.52	2000	HSC	88/10,000	Khawar et al. (2022)
Zn-Co-Mo with rGO	Hydrothermal	Nanosheet	6 M KOH	1,189	1	5.23	7,500	ASSC	95/1,000	Liu et al. (2023)
ZnO/Co_3_O_4_	Hydrothermal	Nanorod	1 M KOH	1,135	1	47.7	7,500	ASSC	83/5,000	Gao et al. (2018)
ZnCo_2_O_4_/rGO/NiO	Hydrothermal	Nanowire	6 M KOH	1,256	3	62.8	7,492.5	SCs	80/3,000	Sahoo and Shim (2017)
ZnO @CoMoO_4_	Hydrothermal	Nanorod-core shell	2 M KOH	1.57 F/cm^2^	0.002 A/cm^2^	—	—	SCs	109/5,000	Cao et al. (2016)
Co_3_O_4_/GO	Chemical bath deposition	Flaky	1 M KOH	1,166.4	2	243	16.2	ASSC	—	Alshoaibi et al. (2022)
MnO_2_/GO	Chemical bath deposition	Circular	1 M KOH	699	2	303.6	20.6	ASSC	—	Alshoaibi et al. (2022)
NiO/GO	Chemical bath deposition	Rectangular rod	1 M KOH	1,032	2	153	10.4	ASSC	—	Alshoaibi et al. (2022)
Co_3_O_4_/MnO_2_/NiO-GO	Chemical bath deposition	Spherical shape	1 M KOH	2,482	2	334.8	32.2	ASSC	96.5/5,000	Alshoaibi et al. (2022)

## 5 Application of metal oxide-based electrode materials

SCs are the primary power source in electric cars and hybrid transportation since they have been employed as short-term energy storage for progressive braking (Şahin et al., 2022). The main problem for all transportation systems is the production of CO_2_, which leads to more energy consumption, which can be solved by employing SCs that provide a large amount of carbon-free energy.

**FIGURE 22:**
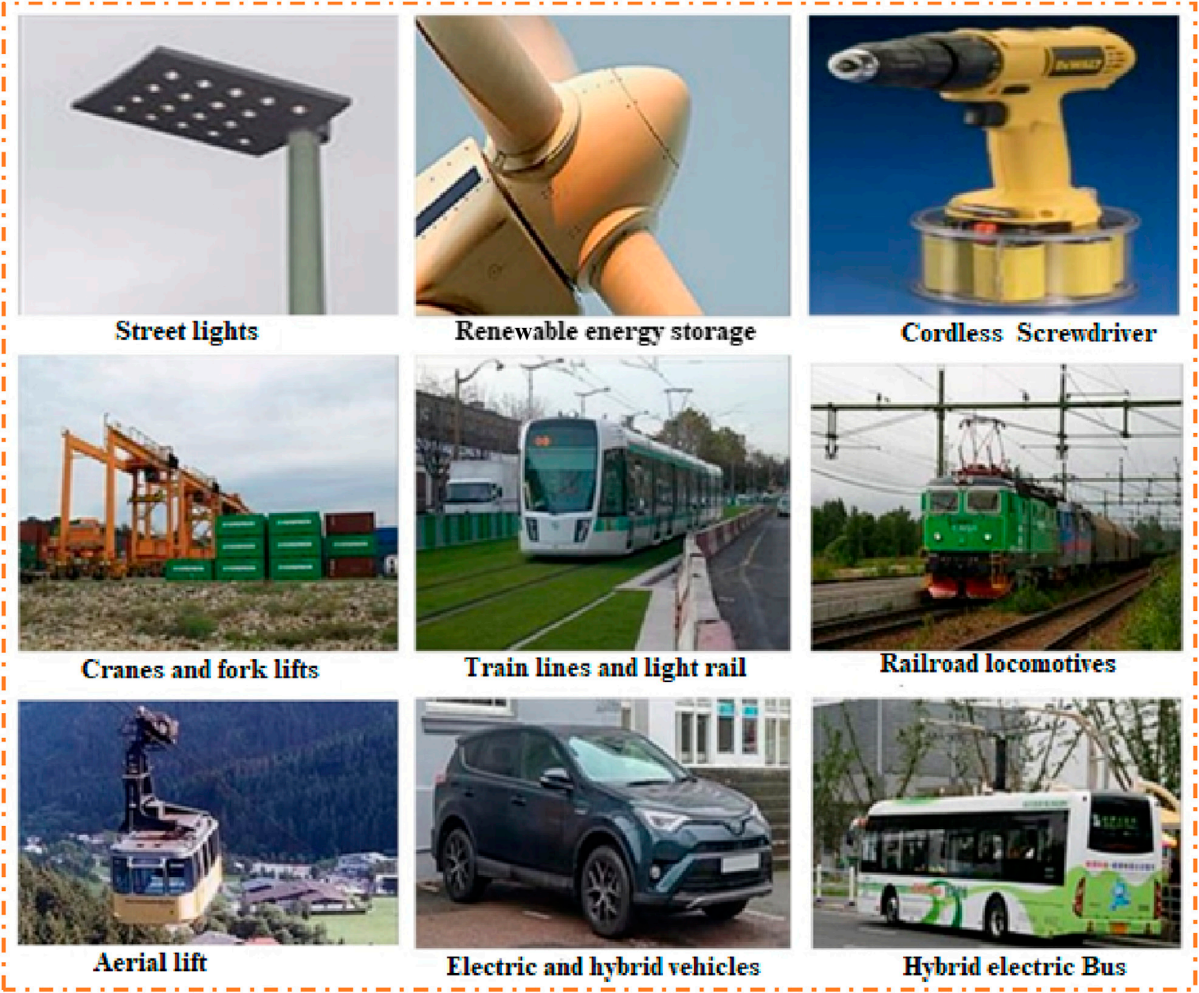
Main examples of supercapacitor applications. Reprinted with permission from Şahin et al. (2022).

Figure 22 shows the uses of SCs in various places, like street lights, renewable energy sources, cordless screwdrivers, and transportation systems such as cranes, train lines, railroads, aerial lifts, and electric and hybrid cars. In all forms of transportation systems, the machine element requires a quick storage of energy and a quick transmission of power, both of which can be provided by SCs. MO-based supercapacitors have a high specific capacitance, quick charge transfer, and cycle stability, making them ideal for applications that require both high power and energy storage capacities. It has been proposed that SCs, as compared to other energy storage devices, are a replacement alternative for single and hybrid applications. Ongoing research and initiatives attempt to improve the ED of SCs and identify novel electrode materials to increase their potential use in EESDs (Şahin et al., 2022).

**FIGURE 23:**
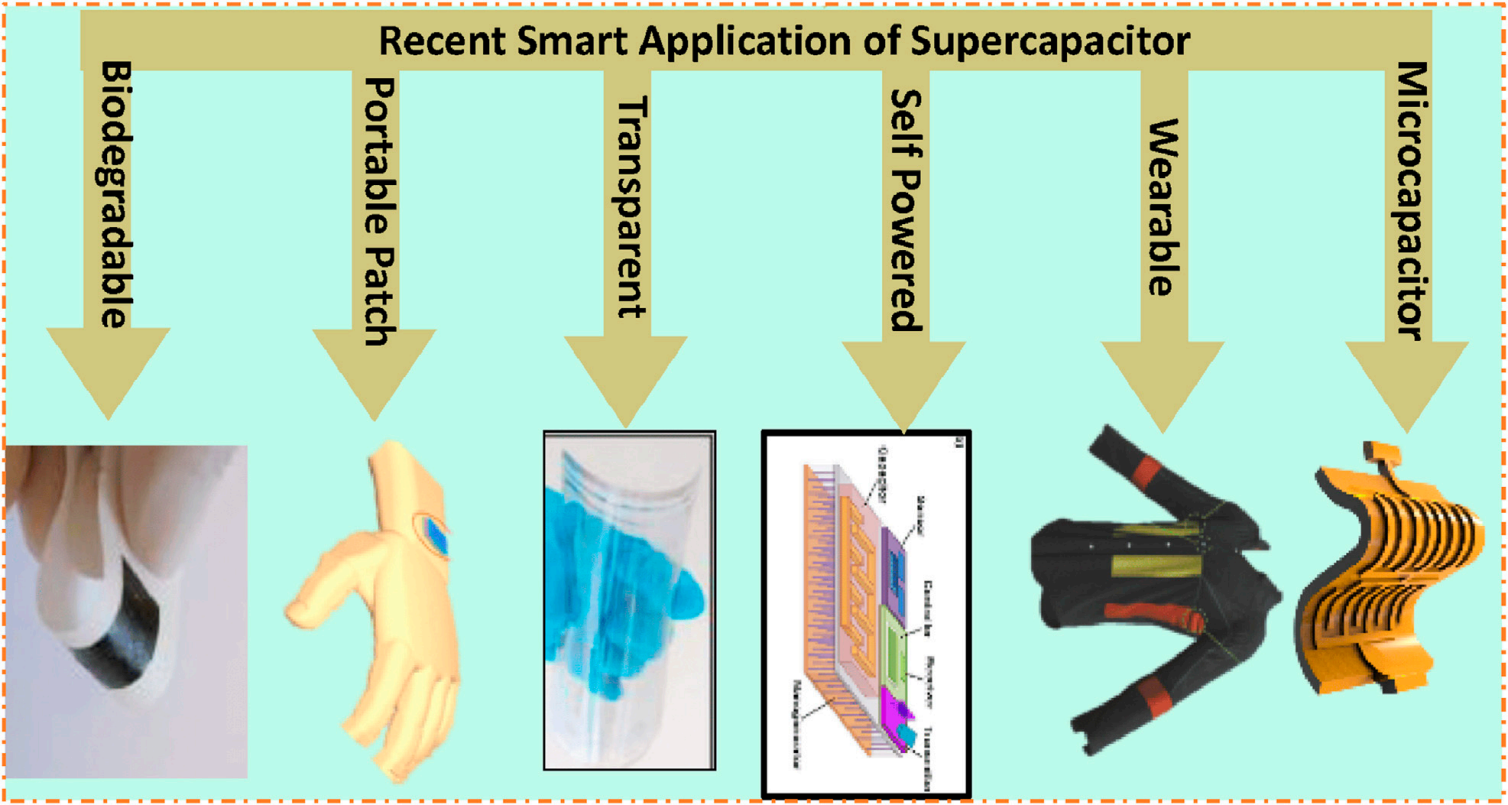
Commercial progress of MOs and their composite-based electrode materials in smart technology. Reprinted with permission from Kumar N. et al. (2022).

MOs and their composite-based electrode materials exhibit several properties, such as high-power conversion efficiencies, tunable optical and electrical properties, excellent charge transport properties, fast charging capability, high sensitivity to changes in environmental conditions, and varying resistive properties, making them a good candidate for supercapacitor applications. Due to the presence of these distinguished properties, metal oxides and their composites can be used in various places, like portable electronics (e.g., smartphones, tablets, and laptops), hybrid and electric cars for renewable energy storage, wearable electronics, energy harvesting systems, and medical equipment (e.g., implants and wearable health monitors) (Schneuwly, 2009; Bottu et al., 2012; Kuperman et al., 2012; Tabish, 2018). Figure 23 shows the smart application of MOs and their composite-based electrode materials in supercapacitor technology. In the near future, hybrid technologies may soon be able to handle energy storage issues.

Supercapacitors are an exciting development with numerous applications. Yet significant obstacles remain related to their advancement, such as choosing an appropriate electrode and electrolyte to obtain high energy density because the electrode materials and electrolytes limit performance.

Continuous investigation into existing device technology, as well as the motivation for novel concepts, has resulted in several breakthroughs, and the launch of a suitable energy device is necessary to propel it to new frontiers. In this respect, it is predicted that qualities like excellent-power density, quicker charging rate, and upgraded stability of storage systems for safe, sustainable energy applications that encompass a broad range of energy demands can be obtained in the near future (Kumar N. et al., 2022).

## 6 Conclusion and outlook

In conclusion, it is observed that researchers are emphasizing on sources of clean energy in order to meet the world’s energy problems. These energy sources depend on efficient and sustainable methods for storing energy to provide reliable, consistent, affordable, safe, and sustainable energy production. The most significant barrier to the extensive and successful use of renewable energy sources is the availability of cost-effective energy storage technologies. From this point of view, this review generalized the present progress in several preparation methods, the morphology of the electrode materials, and the electrochemical performance of MO electrode materials for SC applications. Additionally, it presented a comprehensive overview of the types of SCs and MOs and their composites with carbon, as well as conducting polymer-based electrode materials for SC application. Despite the fact that many years have been devoted to studying the efficient, inexpensive, and rapid synthesis of MO nanoparticles, sustainable synthesis methods have become crucial for bringing down the cost of materials and equipment in a way that benefits both industries and the environment. From this point of view, nanostructured metal oxide and their composites (mono/bi/tri-metal oxide and composites of oxide with conducting polymers and carbon-based electrode materials) are highly favorable for SC applications due to the presence of synergistic effects in the hybrid materials, which are rich in redox activity, excellent conductivity, and chemical stability, which makes them excellent for SC applications. MO’s electrode materials possess high specific capacitance due to the presence of more active sites for redox reactions, which allows them to save a substantial quantity of charge per unit mass. Additionally, the variable features of MO electrode materials can be obtained through the optimization of composition, structure, and morphology, which help attain particular criteria for SCs like specific capacitance, cyclic stability, and capability. This tunability of MO-based electrode materials can maximize charge storage while also improving ion transport and kinetics, which provide a level of precision compared to other kinds of electrode materials, which may struggle to achieve. In all this research, it has been suggested that, compared to simple metal oxides (mono/bi/tri-metal oxides), composites of MOs with carbon have more distributed redox-active sites and a larger surface area, resulting in reduced electrical resistivity, increased redox current, and improved electrochemistry.

It is expected that greater study and advancement in the field of energy storage systems would increase the appropriateness and efficiency of SCs for a variety of energy storage systems.

1. It has been determined that the performance of SCs mainly depends on synthesis methods, the physical as well as chemical types of the materials at the electrode, and the electrolytes employed in the device. By selecting appropriate synthesis processes, we may strengthen the electroactive sites for storing energy effectively, as well as improve the shape, size, and surface of the electrode, which can increase the electrochemical presentation of SCs.
2. Understanding surface chemistry will help establish a better interfacial relation between electrode and electrolyte materials, which will result in an outstanding presentation of SCs. NP materials may significantly improve electrochemical performance, such as ED, PD, cycle stability, ionic conductivity, and proficiency rate, which are essential for employing SCs.
3. It has been noted that the presentation of the electrode material can be enhanced by introducing novel materials in SCs, which must have the following properties: excellent electrical conductivity, varying and multiple states of oxidation, providing a large surface area for oxidation and reduction reactions, forming composites through doping, having high potential windows for operation, and using 3D NMs with large surface areas. A noteworthy effort should be made to develop novel electrode materials for electrochemical production of SCs.
4. Hybrid devices that combine batteries and supercapacitors are the best options for powering the next generation of mobile electronics and other devices. This setup allows compact rechargeable components to be integrated into small electrical items like wristwatches, sensors, mobile phones, headphones, and numerous others.

However, researchers are working to enhance ESD by increasing ED and PD, and one of the fascinating aspects is the use of hybrid technology. Furthermore, it has been observed that the majority of researchers are focusing on the composite of metal oxide with carbon-based materials, metal phosphates, metal phosphides, and metal sulfides, as well as metal organic frameworks, which may be useful in obtaining the required electrode materials.
